# A novel cathode interphase formation methodology by preferential adsorption of a borate-based electrolyte additive

**DOI:** 10.1093/nsr/nwae219

**Published:** 2024-06-25

**Authors:** Danfeng Zhang, Jiabin Ma, Chen Zhang, Ming Liu, Ke Yang, Yuhang Li, Xing Cheng, Ziqiang Wang, Huiqi Wang, Wei Lv, Yan-Bing He, Feiyu Kang

**Affiliations:** Institute of Materials Research (IMR), Tsinghua Shenzhen International Graduate School, Tsinghua University, Shenzhen 518055, China; Institute of Materials Research (IMR), Tsinghua Shenzhen International Graduate School, Tsinghua University, Shenzhen 518055, China; Institute of Materials Research (IMR), Tsinghua Shenzhen International Graduate School, Tsinghua University, Shenzhen 518055, China; Institute of Materials Research (IMR), Tsinghua Shenzhen International Graduate School, Tsinghua University, Shenzhen 518055, China; Institute of Materials Research (IMR), Tsinghua Shenzhen International Graduate School, Tsinghua University, Shenzhen 518055, China; Institute of Materials Research (IMR), Tsinghua Shenzhen International Graduate School, Tsinghua University, Shenzhen 518055, China; Institute of Materials Research (IMR), Tsinghua Shenzhen International Graduate School, Tsinghua University, Shenzhen 518055, China; Institute of Materials Research (IMR), Tsinghua Shenzhen International Graduate School, Tsinghua University, Shenzhen 518055, China; School of Material Science and Engineering & School of Energy and Power Engineering, North University of China, Taiyuan 030051, China; Institute of Materials Research (IMR), Tsinghua Shenzhen International Graduate School, Tsinghua University, Shenzhen 518055, China; Institute of Materials Research (IMR), Tsinghua Shenzhen International Graduate School, Tsinghua University, Shenzhen 518055, China; Institute of Materials Research (IMR), Tsinghua Shenzhen International Graduate School, Tsinghua University, Shenzhen 518055, China

**Keywords:** Ni-rich layered oxides cathode, preferential adsorption, electrolyte additives, interfacial stability, high-energy-density pouch cells

## Abstract

The coupling of high-capacity cathodes and lithium metal anodes promises to be the next generation of high-energy-density batteries. However, the fast-structural degradations of the cathode and anode challenge their practical application. Herein, we synthesize an electrolyte additive, tris(2,2,3,3,3-pentafluoropropyl) borane (TPFPB), for ultra-stable lithium (Li) metal||Ni-rich layered oxide batteries. It can be preferentially adsorbed on the cathode surface to form a stable (B and F)-rich cathode electrolyte interface film, which greatly suppresses the electrolyte-cathode side reactions and improves the stability of the cathode. In addition, the electrophilicity of B atoms in TPFPB enhances the solubility of LiNO_3_ by 30 times in ester electrolyte to significantly improve the stability of the Li metal anode. Thus, the Li||Ni-rich layered oxide full batteries using TPFPB show high stability and an ultralong cycle life (up to 1500 cycles), which also present excellent performance even under high voltage (4.8 V), high areal mass loading (30 mg cm^−2^) and wide temperature range (−30∼60°C). The Li||LiNi_0.9_Co_0.05_Mn_0.05_O_2_ (NCM90) pouch cell using TPFPB with a capacity of 3.1 Ah reaches a high energy density of 420 Wh kg^−1^ at 0.1 C and presents outstanding cycling performance.

## Teaser

Boron-based electrolyte additives are preferentially adsorbed on cathode surface to form a stable cathode electrolyte interface, which greatly suppresses side reactions of electrolyte with high-voltage cathode and improves their stability.

## INTRODUCTION

Safe and sustainable high-energy-density Li batteries are essential for the further development of electric vehicles and energy storage, which drives the exploration of a high-capacity cathode to couple with the Li metal anode (LMA) [1–3]. Among various cathodes, Ni-rich layered oxides (LiNi*_x_*Co*_y_*Mn_1−_*_x_*_−_*_y_*O_2_, *x* ≥ 0.8, NCM) with high Ni content have emerged as promising candidates due to their high discharge capacity (>200 mAh g^−1^), high output voltage (3.8 V) and low cost [4,5]. However, the high Ni content leads to severe side reactions with electrolytes because of the highly reactive Ni^4+^ species in delithiated cathodes [6,7], which causes the bulk and surface phase transformation [8,9], over-growth of cathode electrolyte interphase (CEI) [10–12] and transition metal (TM) dissolution [13,14]. Moreover, the dissolved TM ions can migrate to the Li metal anode side and to be deposited on LMA, which would damage the solid electrolyte interphase (SEI) and increase the resistance and consume both the electrolyte and Li metal [10]. These issues lead to fast battery failure. Therefore, it is a huge challenge to achieve high performance and stability of LMA||NCM batteries under realistic conditions, such as high-loading cathode (typical industry level is >3.5 mAh cm^−2^), low negative to positive (N/P) ratio, lean electrolyte (∼2–5 g (Ah)^−1^) and high cut-off voltages [15–18].

Various strategies have been investigated to address these issues, including heteroatom doping to stabilize the crystal structure [19–21] and constructing an inert CEI or coating to prevent side reactions [13,16,22,23]. For example, the Li_1.4_Y_0.4_Ti_1.6_PO_4_ [24] and inert Al_2_O_3_ [25] coating layers have been utilized to stabilize the structure of Ni-rich NCM through alleviating stress accumulation and suppressing surface side reactions, but they are easily exfoliated from bulk materials upon cycling due to weak interaction, high lattice mismatch, and dramatic anisotropic lattice contraction along the crystallographic *c*-axis [26]. Although the heteroatom doping is a simple and important strategy to improve structure stability, the doping process is usually conducted under high temperatures and potentially leads to element segregation. Compared with the above methods, electrolyte engineering is a more promising strategy that is easy to implement in practice. However, commercial carbonate electrolytes cannot provide sufficient (electro)chemical stability for Ni-rich cathodes due to their high catalytic activity that induces the electrolyte solvents oxidation [13,27,28]. Using high- or localized high-concentration electrolytes and fluorinated-based electrolytes can help form a stable SEI or CEI to improve the cycling stability of LMA||NCM batteries [29,30]. Nevertheless, these electrolytes face some obstacles for commercialization, such as poor wettability, low ionic conductivity and high cost [31–33]. Introducing electrolyte additives to carbonate electrolytes is a more feasible and effective way to ameliorate cycling performance [34]. For instance, the sacrificial additives such as lithium difluorophosphate [13] and lithium difluoro(oxalato)borate [35] can construct robust CEI films. The electrolyte additives containing silane derivatives (Si-O [36], Si-N [22,37], P-N [38]) and isocyanate groups could eliminate hydrogen fluoride and water to suppress the transition metal dissolution [39]. However, the conventional CEI is usually formed by simultaneous catalytic oxidation reaction of electrolytes and their additives, which does not sufficiently withstand the electrolyte from sustained oxidation under high voltage and high-loading cathode [40] due to the porous and mechanically weak CEI oxidized by the solvent [41].

Herein, we synthesize an electrolyte additive, tris(2,2,3,3,3-pentafluoropropyl) borane (TPFPB), featuring borate functional groups and high F concentration pentafluopropyl groups, which can be preferentially adsorbed on the NCM surface to greatly suppress the severe catalytic reactions between NCM and carbonate-based electrolytes. TPFPB not only helps form a B- and F-rich CEI with high mechanical strength and Li^+^ transport kinetics to prevent the Li salt and solvent decomposition on the cathode side, but also can enhance the solubility of LiNO_3_ 30 times higher in the ester electrolyte to significantly improve the stability of LMA. Thus, the assembled Li||LiNi_0.8_Co_0.1_Mn_0.1_O_2_ (NCM811) batteries show excellent cycling stability at harsh operation conditions such as high voltage (4.8 V), high areal loading (30 mg cm^−2^), and wide operation temperature (−30∼60°C). The TPFPB additive also works well with the NCM90 cathode to assemble a Li||NCM90 pouch cell with a high energy density of 420.96 Wh kg^−1^. This work provides a novel strategy that an electrolyte additive can be preferentially adsorbed on the cathode to construct a robust CEI, suggesting a brand-new direction for electrolyte additive design for a stable high voltage cathode.

## RESULTS AND DISCUSSION

### Design and synthesis of TPFPB

**Figure 1.**
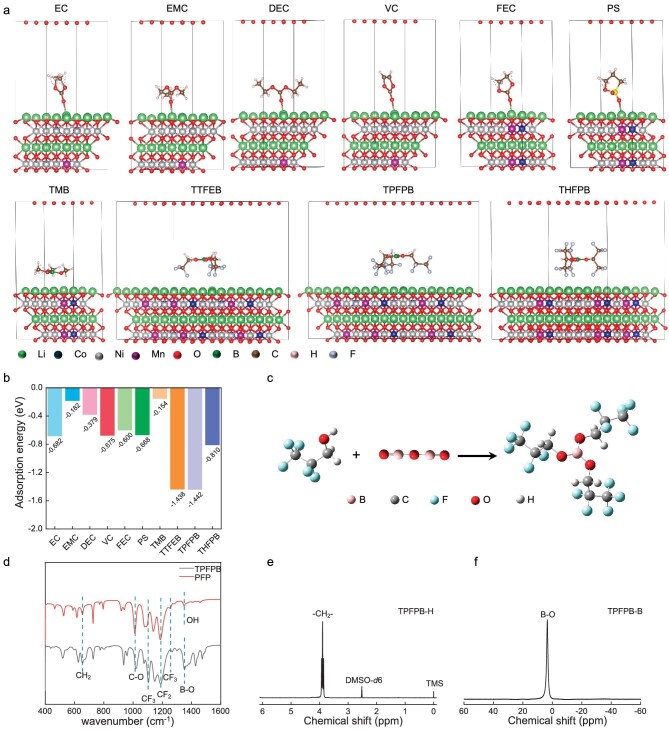
Design and synthesis of TPFPB. (a) Scheme of adsorption status for different components on NCM811 surface. (b) Adsorption energy values of different components on NCM811. (c) Scheme of synthesis of TPFPB. (d) FTIR of TPFPB and PFP. (e) ^1^H NMR and (f) ^11^B NMR of TPFPB.

Due to the electronegativity of fluorine, the higher fluorine content in the solvent molecules usually leads to a greater adsorption capacity on the NCM cathode, but if the steric hindrance of the molecule is too large, the rotation of the molecule will be restricted, thereby reducing the adsorption energy between them. The density functional theory (DFT) calculations reveal the highest adsorption energy of TPFPB (−1.442 eV) on the NCM811 surface among the different electrolyte solvents, including ethylene carbonate (EC, −0.682 eV), diethyl carbonate (DEC, −0.379 eV), ethyl methyl carbonate (EMC, −0.182 eV) and vinylene carbonate (VC, −0.675 eV). It also shows much higher adsorption energy compared to commercial electrolyte additives such as 1,3-propane sultone (PS, −0.668 eV), 4-fluoroethylene carbonate (FEC, −0.600 eV) and other borate esters (trimethyl borate (TMB, −0.154 eV), tris(2,2,2-trifluoroethyl) borate (TTFEB, −1.438 eV) and tris(1,1,1,3,3,3-hexafluoroisopropyl) borate (THFPB, −0.810 eV)). These results suggest that the TPFPB could inhibit solvents from being adsorbed and oxidized on the NCM811 surface to form polymeric components-dominated CEI with inferior stability and high ion diffusion resistance (Fig. 1a, b).

TPFPB was synthesized by the condensation reaction of B_2_O_3_ with pentafluoro-1-propanol (PFP) at 80°C (Fig. 1c). The sp^2^ boron has an electron-deficient nature that enables it to coordinate with the PO_4_^−^ anion and form a polyanionic group, which significantly increases the electrochemical stability window of TPFPB [42]. The fluorine groups contribute to the formation of LiF-rich CEI and SEI. The Fourier transform infrared spectroscopy (FTIR) spectra show that the C−O peaks of TPFPB (1021 cm^−1^) shift to higher wavenumbers compared with those of PFP (1012 cm^−1^) while the other peaks (CF_2_, CF_3_) remain unchanged, indicating the electrophilic effect of the B atom (Fig. 1d). The ^1^H NMR of TPFPB reveals the chemical shift centered at 3.90 ppm, corresponding to -CH_2_ (Fig. 1e). The ^13^C NMR spectra display multiple peaks in the range of 111–124 ppm, corresponding to the −CF_3_ and −CF_2_- groups [43] (Fig. S1a), which can be further confirmed by the ^19^F NMR (Fig. S1b). The singlet peak located at 3.41 ppm in ^11^B NMR spectra of TPFPB can be assigned to the B−O bond (Fig. 1f). The ^13^C and ^19^F NMR spectra of PFP are identical to those of TPFPB, verifying the presence of the pentafluopropyl group in TPFPB (Fig. S2). The density functional theory (DFT) calculations present that the highest occupied molecular orbital (HOMO) level of the TPFPB is −9.317 eV, which is lower than the other solvent components of the electrolyte (Fig S3a), indicating that the TPFPB is difficult to be oxidized. The linear sweep voltammetry curves confirm that the electrochemical window of TPFPB-containing electrolyte is similar to BE at the critical current density of 5 μA cm^−2^ (Fig. S3b).

### Electrochemical performance of Li||NCM811 cells with TPFPB

**Figure 2.**
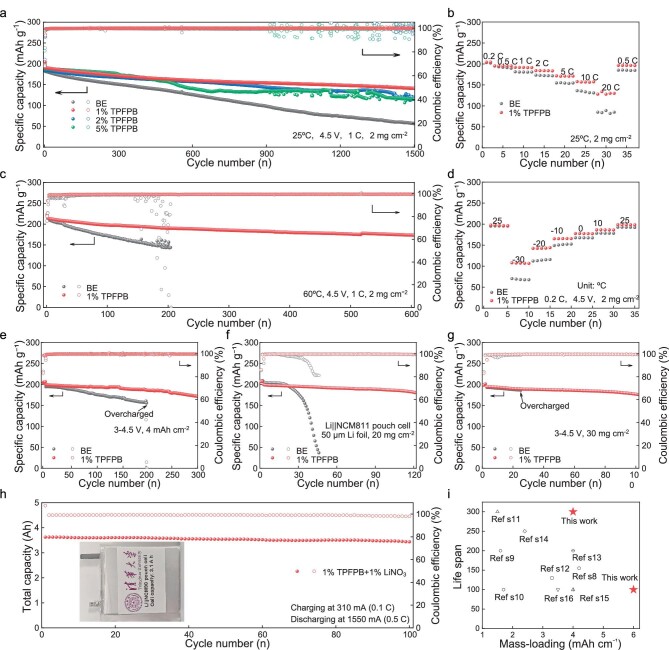
Electrochemical performance of Li||NCM811 cells using electrolytes containing different amounts of TPFPB. (a) Cycling performance at 25°C. (b) Rate performance at 25°C. (c) Cycling performance at 60°C. (d) Capacity at 0.2 C between −30 and 25°C. (e) Cycling performance at 25°C between 3 and 4.5 V, cathode loading: ∼20 mg cm^−2^, 200 μm Li foil, charge/discharge rate: 0.1/0.333 C. (f) Cycling performance of single-layer pouch cells at 25°C between 3 and 4.5 V, cathode loading: ∼20 mg cm^−2^, 50 μm Li foil. (g) Cycling performance at 25°C between 3 and 4.5 V, cathode loading: ∼30 mg cm^−2^, 200 μm Li foil. Charge/discharge rate is 0.1/0.333 C. (h) Cycling performance of pouch cell at 25°C between 3 and 4.3 V, charge/discharge rate: 0.1/0.5 C (the inset is the optical graph of the pouch cell). (i) Electrochemical performance comparison with recently reported state-of-the-art electrolytes.

The assembled Li||NCM811 batteries with electrolytes containing different TPFPB contents were characterized (active material loading: ∼2 mg cm^−2^) between 3 V and 4.5 V at different temperatures. As shown in Fig. 2a, the Li||NCM811 cell using BE with 1%TPFPB at 25°C shows a high-capacity retention of 74.80% after 1500 cycles at 1 C, which is much higher than that of the Li||NCM811 cell (32.91%) using the basic carbonate electrolyte without TPFPB (BE) and the electrolytes containing 0.5% and 2%TPFPB (63.82 and 64.71%, respectively). The cell using BE with 1%TPFPB also shows excellent rate performance with high specific discharge capacities of 156.9 and 130 mAh g^−1^ at 10 and 20 C, respectively, much higher than the other cells (Fig. 2b). The improved rate performance can be ascribed to its much lower electrochemical impedance (Fig. S4). Notably, the Li||NCM811 cell using BE with 1%TPFPB also shows high-capacity retentions of 81.24% at 60°C after 600 cycles and 93.50% at −20°C after 300 cycles (Fig. 2c, Fig. S5). In contrast, the Li||NCM811 cell using BE shows much lower capacity retentions of 67.80% after only 200 cycles at 60°C and 91.06% after 300 cycles at −20°C (Fig. 2c, Fig. S5). At a lower temperature of −30°C (Fig. 2d), the Li||NCM811 cell using BE with 1%TPFPB delivers a high specific capacity of 108.0 mAh g^−1^ (0.2 C), 54.6% of the room temperature (RT) capacity, much higher than that of the Li||NCM811 cell using BE (68.2 mAh g^−1^, 35.4% of the RT capacity). In addition, the capacity of the Li||NCM811 cell using BE with 1%TPFPB is fully recovered when the temperature returns to RT, while that of the Li||NCM811 cell using BE is 98.6% (Fig. S6), suggesting the excellent stability of the Li||NCM811 cell using BE with 1%TPFPB. The cycle performance enhancement of Li||NCM811 cells using BE with 1%TPFPB may be attributed to the much lower electrochemical impedance under −30°C (Figs S7 and S8). The ionic conductivity trend at different temperature was fitted by the Vogel–Tammann–Fulcher (VTF) equation. The activation energy of BE with 1%TPFPB is 1.84 kJ mol^−1^, which is much smaller than that of BE (5.42 kJ mol^−1^). Therefore, the BE with 1%TPFPB more easily transports Li ions at low temperature to reduce the charge and discharge polarization.

The use of a high-loading cathode and thin LMA is a prerequisite to achieve the high energy density of Li metal batteries, which presents great difficulty in achieving satisfactory cycling stability. Notably, the cell using BE with 1%TPFPB with a high-loading NCM811 cathode (∼20 mg cm^−2^, ∼4 mAh cm^−2^) and thin Li foil (200 μm, ∼40 mAh cm^−2^) shows good cycling stability with high-capacity retention of 85.8% after 300 cycles. In contrast, the battery using BE is overcharged after ∼200 cycles (Fig. 2e). The discharge specific capacity of Li||NCM811 cells with high mass loading of 20 mg cm^−2^ at 1 C is 169.3 mAh g^−1^, which is much higher than that of the Li||NCM811 cells using BE (140.1 mAh g^−1^ at 1 C, Fig. S9). The good rate performance of Li||NCM811 cells using BE with 1%TPFPB may be attributed to the high ionic conductivity of BE with 1%TPFPB and low CEI resistance. For the single-layer pouch cell with limited electrolyte (E/C ratio of ∼5 g (Ah)^−1^), the TPFPB additive enables high-capacity retention of 90.32% after 120 cycles of the battery, in contrast to rapid capacity degradation of the battery using BE after 20 cycles (Fig. 2f). Under a higher cathode mass-loading of ∼30 mg cm^−2^ (6 mAh cm^−2^), the cell using BE with 1%TPFPB also achieves a high-capacity retention of up to 90.56% after 100 cycles, while those of BE overcharged within 26 cycles (Fig. 2g). Our results show the highest cathode mass-loading and lifespan in coin cells compared with recently reported state-of-the-art batteries (Fig. 2i, Table S1). With a higher cut-off voltage of 4.8 V, the battery using BE with 1%TPFPB still shows a high capacity retention of 83.65% after 100 cycles (Fig. S10). Furthermore, the TPFPB was also beneficial in improving the electrochemical performance of LiNi_0.9_Co_0.05_Mn_0.05_O_2_ (NCM90) and LiCoO_2_ (LCO) cathode during high voltage cycling (Fig. S11). The above results clearly prove that the TPFPB additive effectively enhances the cycling stability of the batteries even under practical conditions.

Also, the addition of TPFPB with electron-withdrawing properties appears to serve as an anion receptor to attract NO_3_^−^ under moderate heating temperatures [44], which enhances the solubility of LiNO_3_ in the carbonate electrolyte by 30 times from ∼0.012 M [45] to 0.368 M (Fig. S12). The solubility enhancement of LiNO_3_ in ester electrolytes could significantly improve the deposition behavior of Li metal and inhibit the generation of Li dendrites even at high Li deposition of 4 mAh cm^−2^ (Fig. S13). This can further improve the cycling stability of the battery, even with the NCM90 cathode (Fig. S14). The Li||NCM90 cells using BE with 1%TPFPB and 1%LiNO_3_ show a very high-capacity retention of 96.34% after 100 cycles at 0.5 C charging and 1 C discharging, which could be ascribed to the improved structural stability of the NCM90 cathode and suppressed Li dendrite growth of LMA (Figs S15, S16). To further verify the practicality, pouch cells with a capacity of 3.1 Ah were fabricated, which show an ultrahigh energy density of 420.96 Wh kg^−1^ under a discharging rate of 0.1 C. Such a cell also shows excellent stability of 100 cycles with 94.88% capacity retention under the charge/discharge rate of 0.1/0.5 C (Fig. 2h). Our results show that the electrolyte design is critical in realizing high-energy-density LMBs.

### Effects of TPFPB on the NCM811 cathode

**Figure 3.**
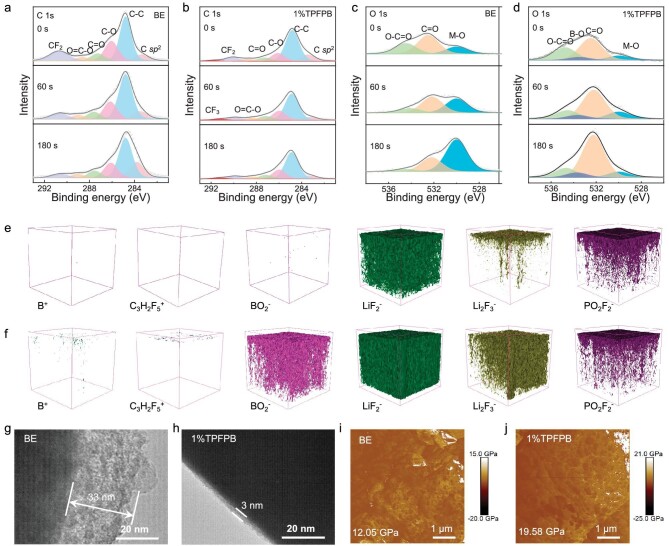
CEI analysis of NCM811 cathode. *Ex situ* XPS measurements and analysis of NCM811 cathode retrieved from (a, c) Li||NCM811 cells using BE and (b, d) BE with 1%TPFPB after 5 cycles at 0.1 C under 25°C, (a, b) C 1*s*, (c, d) O 1*s*. (e, f) *Ex situ* TOF-SIMS 3D reconstruction of the sputtered volume on the cycled NCM811 surface in (e) BE and (f) BE with 1%TPFPB. *Ex situ* TEM images of NCM cathode retrieved from Li||NCM811 cells using (g) BE and (h) BE with 1%TPFPB after 100 cycles at 25°C, the charging/discharging rate: 0.1/0.333 C. Young's modulus of CEI of (i) Li||NCM811 cells using BE and (j) Li||NCM811 cells using BE with 1%TPFPB after 5 cycles at 0.1 C under 25°C. The cells were disassembled at a fully discharged state.

X-ray photoelectron spectroscopy (XPS) was conducted to analyze the chemical composition of the CEI on NCM811 after 5 cycles under 25°C. Compared with the C 1s spectra on the depth profiling of the NCM811 cycled in BE, the NCM811 cycled with TPFPB additives show a peak at 291.4 eV corresponding to CF_3_. In O 1s spectra, an additional B-O peak (533.5 eV) can also be observed. The F 1s spectra suggest a higher LiF content and a lower P-F content in CEI formed in the BE with 1%TPFPB (Fig. S17). These results suggest that the TPFPB is decomposed on the NCM811 surface to participate in CEI formation (Fig. 3a–d). Due to overlapping of the spectral range of B 1s from TPFPB and P 2 s from LiPF_6_ salt, this interferes with the analysis of the composition of CEI with TPFPB. Therefore, the LiClO_4_ salt was used to assemble another Li||NCM811 battery. As shown in Fig. S18, the B-O peak (190.0 eV) appears on the cathode cycled with BE with 1%TPFPB using LiClO_4_ salt. The XPS in-depth atomic percentage profile of NCM811 cathode retrieved from Li||NCM811 cell using BE shows much higher carbon content than those of Li||NCM811 cells using BE with 1%TPFPB, indicating that solvent decomposition has been suppressed due to the preferential adsorption of TPFPB on the cathode surface (Fig. S19). Time-of-flight secondary ion mass spectrometry (TOF-SIMS) measurements show that the interphase products, including the boron species (represented by B^+^ and BO_2_^−^) and fluorine species (represented by C_3_H_2_F_5_^+^, LiF_2_^−^ and Li_2_F_3_^−^), appear in the CEI using BE with 1%TPFPB. The B^+^, BO_2_^−^ and C_3_H_2_F_5_^+^ species should be derived from the decomposition of TPFPB. The PO_2_F_2_^−^ species on the NCM811 surface cycled with TPFPB are fewer than those with BE, which is similar to XPS results, indicating that the TPFPB could inhibit LiPF_6_ decomposition (Fig. 3e, f and Fig. S20). The XPS and TOF-SIMS results indicate that the TPFPB could be preferentially adsorbed and oxidized to form a B- and F-rich CEI film to prevent Li salt and solvent decomposition.

The thickness of the CEI on NCM811 after 100 cycles at 25°C was measured by transmission electron microscopy (TEM). The uniform and amorphous CEI with a thickness of ∼3 nm was formed on the surface of cycled NCM811 using BE with 1%TPFPB, which is much thinner than that formed using BE (33 nm) (Fig. 3g, h). In addition, atomic force microscopy (AFM) also shows a higher Young's modulus of the CEI formed using BE with 1%TPFPB (19.58 GPa) due to the higher inorganic content, which is much higher than that using BE (12.05 GPa) (Fig. 3i, j). Thus, the thin and robust CEI can maintain integrity during cycling to suppress the continuous electrolyte decomposition and ensure excellent rate performance and cycling stability.

**Figure 4.**
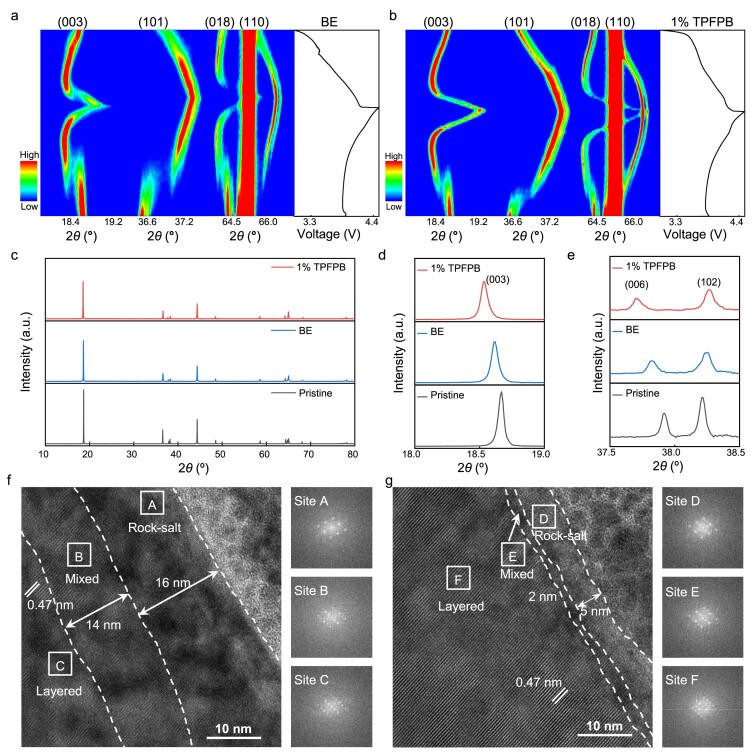
Structure characterizations of NCM811 cathode. Operando XRD characterization for NCM811 cathode during the initial charge-discharge cycles using (a) BE and (b) BE with 1%TPFPB at 0.2 C. (c–e) *Ex situ* XRD measurements of NCM811 cathode before and after 200 cycles using BE and the BE with 1%TPFPB at 1 C under 25°C. *Ex situ* TEM and fast Fourier transform (FFT) images of cycled NCM811 cathodes using (f) BE and (g) BE with 1%TPFPB after 100 cycles at 1 C under 25°C. The cells were disassembled at fully discharged state.

The TPFPB-derived inorganic-rich CEI could also suppress the phase changes of NCM811. Operando X-ray diffraction (XRD) experiments show the structural changes of NCM811 during the initial cycle between 3.0 and 4.5 V (Fig. 4a, b). The NCM811 undergoes an H1-M-H2-H3 phase transition during the charge-discharge process. It is shown that the H1-M phase transition of NCM811 using BE with 1%TPFPB is effectively suppressed compared with that using BE. The parameter *c* for NCM811 cathode slightly increases after the first cycle due to the initial nonequilibrium ‘two-phase’ behavior [46]. The *ex-situ* XRD patterns show that the peaks corresponding to the (003) and (006) crystal interplanar of NCM811 cycled in BE with 1%TPFPB shift to a lower degree (Fig. 4c–e), indicating the larger Li slab space that can promote Li ion transport [47]. The TEM images show a thin rock-salt phase (5 nm) and mixed-phase (2 nm), indicating that the surface reconstruction has been effectively suppressed. In contrast, a much thicker rock-salt phase (16 nm) and mixed-phase (14 nm) is observed on the surface region of the NCM811 particle cycled in BE (Fig. 4f, g). The more severe phase transitions always occur in conjunction and generate more severe lattice strain, which induces micro-cracks and particle breakdown that decreases the cycling stability. The thin and robust CEI formed by TPFPB on NCM811 contains high content of LiF and LiBO_2_, which could inhibit electrolyte corrosion of the cathode. In addition, the formed inorganic-rich CEI by TPFPB could efficiently block the electrolyte penetration into NCM811 to decrease the side reaction between cathode and electrolyte, which can also suppress the phase transformation of NCM811 during long cycling. Thus, the cycled NCM811 cathode with same loading of ∼20 mg cm^−2^ in the BE with 1%TPFPB shows a similar thickness (∼96.4 μm) to the pristine (∼93 μm), and the particles in the cathode remain intact without cracking after 100 cycles (Fig. S21). In sharp contrast, the cathode cycling in BE shows an apparent thickness increase to 117.4 μm, which may be ascribed to the volume expansion of the cracked NCM811 particles. The fracture of particles causes capacity fading and generates newly exposed surfaces, leading to continuous decomposition of the electrolyte during cycling.

### Effects of TPFPB on the LMA

**Figure 5.**
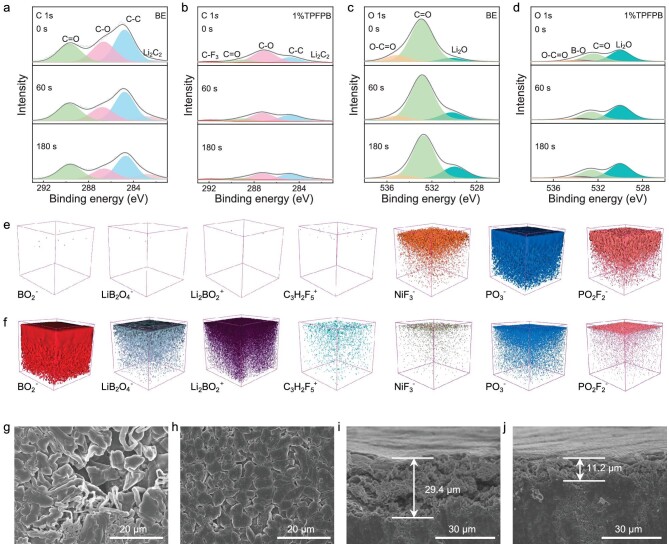
Surface component analysis and morphology of LMA. *Ex situ* XPS measurements and analysis of LMA retrieved from (a, c) Li||NCM811 cells using BE and (b, d) Li||NCM811 cells using BE with 1%TPFPB (a, b) C 1*s*, (c, d) O 1*s*. (e, f) *Ex situ* TOF-SIMS 3D reconstruction of the sputtered volume on the cycled NCM811 surface using BE (e) and BE with 1%TPFPB (f). *Ex situ* SEM characterization of the (g, h) surface morphology and (i, j) cross-section views of the LMA retrieved from the Li||NCM811 cells using (g, i) BE and (h, j) BE with 1%TPFPB after 5 cycles at 0.1 C under 25°C. The cells were disassembled at a fully discharged state.

The formation of stable SEI plays an essential role in ensuring the cycling stability of LMA. Thus, the SEI composition on the LMA surface with different electrolytes was also characterized by in-depth XPS. The peaks corresponding to the CF_3_ and B-O groups suggest that TPFPB also participates in SEI formation (Fig. 5a–d and Fig. S22). In F 1s spectra, the higher ratio of LiF species suggests the TPFPB reduction on LMA, and the lower F-P species content indicates the suppressed LiFP_6_ decomposition (Fig. S23). Figure S24 shows the distribution of selected elements on cycled LMA collected at different depths. The higher content of inorganic components (LiF and Li_2_O) in the SEI formed in the TPFPB electrolyte suggests better stability. The intensity of C 1s and O 1s peaks for LMA cycled in BE is much higher, which shows a strong C=O peak at 289.7 eV [48] and a lower Li content, indicating more organic components in the electrolyte solvent decomposition-dominated SEI (Fig. 5a–d and Fig. S25). The TOF-SIMS also present that more boron species (BO_2_^−^, LiB_2_O_4_^−^ and Li_2_BO_2_^+^) are detected on the LMA surface [49], indicating that the TPFPB is reduced (Fig. 5e–f, Fig. S26). The LiBO_2_ presents a strong absorption to Li^+^ and can greatly improve the interfacial dynamics of Li^+^ migration for homogenous Li nucleation and deposition to form a dense Li layer [50]. In addition, the content of NiF_3_^−^ species on the LMA is also much lower, which is further confirmed by Ni 2p spectra and an inductively coupled plasma optical emission spectrometer (ICP-OES) (Figs S27, S28), suggesting a suppressed TM dissolution of the cathode with TPFPB-derived CEI. As shown in Fig. 5g–h, nonuniform Li deposition and massive Li dendrites are observed on the LMA surface, and the thickness of this layer is 29.4 μm for LMA cycled using BE. In sharp contrast, the dead Li is greatly suppressed in the BE with 1%TPFPB due to formation of a much thinner nonuniform layer (11.2 μm). The dense surface layer is beneficial for reducing SEI growth and suppressing dead Li formation (Fig. 5i–j). Thus, the TPFPB can effectively suppress the formation of Li dendrites, accounting for enhanced cycling performance of LMA.

## CONCLUSION

In conclusion, we develop an electrolyte additive of TPFPB tailored for high voltage Li||NCM811 batteries (4.5 V) with ultra-stable and long-life performance. With only 1% TPFPB in the electrolyte, a high-capacity retention of 74.80% after 1500 cycles at 25°C and 81.24% after 600 cycles at 60°C is achieved. The Li||NCM90 pouch cell using TPFPB (3.1 Ah) reaches high energy density of 420 and 395 Wh kg^−1^ at the discharging rates of 0.1 and 0.5 C, respectively, which show excellent capacity retention of 94.88% after 100 cycles. The dual roles of TPFPB in stabilizing both the cathode and anode are responsible for such excellent performance under practical requirements. The TPFPB is preferentially adsorbed on the surface of NCM811 to participate in forming a thin and robust CEI to reduce the electrolyte decomposition, which can also enhance the solubility of LiNO_3_ in conventional carbonate electrolytes and participates in forming an inorganic-rich SEI on LMA. This research offers valuable insights into an innovative electrolyte design strategy, providing a practical pathway for high-energy-density Li metal batteries.

## METHODS

### General information

Additional details of materials and diversity of employed characterization techniques are presented in the Supporting information.

### Synthesis of tris(2,2,3,3,3-pentafluoropropyl) borane (TPFPB)

A suspension of B_2_O_3_ (23.2 g, 0.333 mol) and pentafluoro-1-propanol (100 g, 0.666 mol) was stirred at 80°C for 24 h. The reaction mixture was purified by distillation to give TPFPB as a clear liquid (50.2 g, 0.109 mol, 49.4%).

### Preparation of TPFTB-containing electrolyte

To prepare electrolyte with 1%TPFPB, 10 μL TPFPB was dissolved in 1 mL BE (1 M LiPF_6_ in EC, EMC and DEC (3:4:3 by volume)) by magnetic stirring at room temperature for 30 min. To prepare electrolyte with 1%TPFPB-1% LiNO_3_, 12 mg LiNO_3_ was added into BE with 1%TPFPB (the density of BE with 1%TPFPB is 1.2 g mL^−1^) and heated to 50°C by magnetic stirring until all the powder was dissolved.

## Supplementary Material

nwae219_Supplemental_File
