# Supplementary material for: A novel cathode interphase formation methodology by preferential adsorption of a borate-based electrolyte additive
Source: Natl Sci Rev. 2024 Jun 25;11(8):nwae219. doi: 10.1093/nsr/nwae219 (PMC11312368; doi:10.1093/nsr/nwae219)
Supplement: nwae219_Supplemental_File [file nwae219_supplemental_file.pdf]

Supplementary Data for

A novel cathode interphase formation methodology by preferential adsorption of a borate-based electrolyte additive

Danfeng Zhang[1], Jiabin Ma[1], Chen Zhang[1], Ming Liu[1], Ke Yang[1], Yuhang Li[1], Xing Cheng[1], Ziqiang Wang[1], Huiqi Wang[2], Wei Lv[1]*, Yan-Bing He[1]*, Feiyu Kang[1]*

[1]Institute of Materials Research (IMR), Tsinghua Shenzhen International Graduate School, Tsinghua University, Shenzhen 518055, China.

[2]School of Material Science and Engineering & School of Energy and Power Engineering, North University of China, Taiyuan 030051, China.

***Corresponding author**. E-mail: lv.wei@sz.tsinghua.edu.cn; he.yanbing@sz.tsinghua.edu.cn; fykang@sz.tsinghua.edu.cn

Materials and Methods

Materials.

The commercial $LiNi_{0.8}Co_{0.1}Mn_{0.1}O_2$ (NCM811, single crystal), $LiNi_{0.9}Co_{0.05}Mn_{0.05}O_2$ (NCM90, multi crystal), $LiCoO_2$ (LCO, multi crystal), polyvinylidene difluoride (PVDF5130), Super P, Al foil, separator (Celgard 2500), and the baseline electrolyte (BE) (1 M $LiPF_6$ in EC, EMC and DEC (3:4:3 by Volume), 1 wt% VC, (H_2O< 20 ppm)) were purchased from Guangdong Canrd New Energy Technology Co., Ltd. B_2O_3 (99%), pentafluoro-1-propanol (PFP, 97%), N-methyl-2-pyrrolidone (NMP, AR) and diethyl carbonate (DEC, 99%, (H_2O< 20 ppm) were purchased from Shanghai Aladdin Biochemical Technology Co., Ltd. Lithium foil (thickness: 200 μm, diameter: 15.6 μm) and single side Lithium coated Cu foil (the thickness of Li and Cu is 50 μm and 10 μm) came from China Energy Lithium Co., Ltd. Dimethyl sulfoxide-*d*6 (DMSO-*d*6, 0.6 mL, 99.9%, +0.03% V/V tetramethyl silane (TMS)) came from Shanghai Macklin Biochemical Technology Co., Ltd. All materials were used as received.

Materials Characterizations.

The X-ray diffraction (XRD) measurements of the samples were carried out on a Rigaku Smartlab with Cu-Kα radiation. The operando XRD test during charging and discharging rate of 18 mA g^{-1} was performed at 25 ℃ and diffraction patterns were collected every 16 min. The morphologies and structures of NCM cathode and Li anode were characterized by a scanning electron microscope (SEM, HITACH S4800) and a Cold-Field-Emission Double Cs-corrected TEM (Thermo Fischer Spectra 300). X-ray photoelectron spectroscopy (XPS) measurements were collected on a PHI 5000 VersaProbe Ⅱ instrument. The ^{19}F, ^{13}C, ^{7}B and ^{1}H nuclear magnetic resonance (NMR) data were collected with NMR spectrometer (ADVANCED Ⅲ 400 MHz, Bruker, Switzerland) with DMSO-*d6* solvent. The transition metal (TM) concentration of the Li anode was measured by an inductively coupled plasma optical emission spectrometer (ICP-OES, SpectroArcosⅡ MV, Germany). The cycled LMA was washed by DEC and dissolved in 2 mL 1 M HCl and then diluted to 50 mL solution with deionized H_2O using a 50 mL volumetric flask, and the TM content on the surface of LMA was calculated by the mass of TM elements in the solution over the mass of active material in the NCM811 cathode. The CEI and SEI component data was collected by time-of-flight secondary ion mass spectrometry (ToF-SIMS, PHI nanoTOF Ⅱ, 30 keV, 2 nA, and the raster size is 60 × 60 μm.). The cross-sectional NCM cathode was investigated by an ion milling system (IMS, HITACH IM4000 plus). Before analysis, the cells were disassembled in an Ar-filled glove box (Mikrouna) (H_2O < 0.1 ppm, O_2 < 0.1 ppm) and the electrode surfaces were rinsed with 5 mL of DMC. After drying at 25 ℃ in glovebox for 10 min, the electrodes were transferred by the transfer sample holder with an Ar-filled to isolate the air.

Fabrication of cells.

Low areal mass loading (~2 mg cm^{-2}) NCM811 cathode was prepared by following steps. The mixture of 0.8 g (80 wt%) NCM811 powder, 0.1 g (10 wt%) PVDF5130 and 0.1 g (10 wt%) Super P was manually ground in an agate mortar for 10 mins in an air environment and then dispersed in 2 mL NMP by magnetic stirring for 4 h to form a slurry. Then the slurry was casted on an Al foil and dried at 120 ℃ for 2 h under vacuum. High areal mass loading (>20 mg cm^{-2}) NCM811, NCM90 and LCO cathode was prepared by following steps. The mixture of 4.7 g (94 wt%) NCM811

(NCM90 or LCO) powder, 0.15 g (3 wt%) PVDF5130 and 0.15 g (3 wt%) Super P was manually ground in an agate mortar for 15 mins in an air environment and then dispersed in 4.1 g NMP by grinding mill for 15 min at 2000 rpm to form a slurry. Then the slurry was casted on an Al foil and dried at 120 ℃ for 2 h under vacuum. The CR2032 Li||NCM811, Li||NCM90 and Li||LCO coin cells were assembled in an Ar-filled glove box (O_2 and H_2O < 0.1 ppm) using 40 μL BE with/without TPFPB. The thickness of lithium foil is 200 μm and the diameter is 15.6 mm. and the thickness of the separator is 25 μm. For single-layer Li||NCM811 pouch cell, NCM811 cathode (mass loading: ~ 20 mg cm^{-2}, 4 cm × 3.5 cm) and one single coated anode (4.3 cm × 4 cm) were stacked one by one and separated by Celgard 2500 (4.5 cm × 4.5 cm), the electrolyte volume is 200 μL. We assembled in a dry room whose dew point is -50 ℃.

Electrochemical measurements.

The electrochemical impedance spectroscopy (EIS) of Li||NCM811cells was performed from 7 MHz to 10 mHz at an amplitude of 5 mV, 6 data points per decade, and the 3.95 V of charging progress (after charge to 3.95 V, a constant-voltage charging was applied until the specific current is lower than 9 mA g^{-1}, and rest for 2 h) applied before carrying out the EIS measurements at 25 ℃ using a VMP3. The Li ion conductivity of BE and BE with 1% TPFPB were test by assembling stainless steel||stainless steel cells under -30~60 ℃ using a VMP3. Cycling and rate performances of Li||NCM811cells were measured at a temperature range of -30~60 ℃ on a battery test system (LAND CT-2001A). Most of the batteries were operated at 25±1 ℃ except part of batteries was tested at 60 ℃, -20 ℃ and -30 ℃. The specific current and specific capacity refers to the mass of the active material in the cathode. The electrochemical energy storage tests at various temperatures were carried out in a constant temperature chamber.

Vogel–Tammann–Fulcher function fitting.

The EIS data of Li||Li cells were collected by VMP3 from -30 to 60 ℃. The VTF behavior[1], which is more relevant for electrolyte organic solutions, is described by equation (1)

$$\sigma = \sigma_0 T^{-\frac{1}{2}} \exp\left(-\frac{B}{T-T_0}\right) \qquad (1)$$

Here B is the pseudo-activation energy for the conductivity (expressed in units of E_a/k), and T_0 is the reference temperature which normally falls 10-50 K below the experimental glass transition, T_g.

Calculations.

***HOMO* and LUMO energy**. The highest occupied molecular orbital and lowest unoccupied molecular orbital (HOMO and LUMO) energy of EC, EMC, DEC, VC, $LiPF_6$ and TPFPB was calculated by quantum-chemical calculations (based on density functional theory, B3LYP/6-311G (d, p)). The optimum structures were optimized by B3LYP method in combination with the 6-31G (d, p) basis set and the more precise energy was calculated by same method in combination with the 6-311 G++ (d, p) basis set. The vibration frequency and intrinsic reaction coordinate (IRC) analysis were adopted to confirm each transition state (TS) that connects both product and reactant in the same pathway at the same level. The stable structure between H_2O and Li^+, PF_6^- and $HDMS^-$ were obtained from the Molecular dynamics (MD) simulation with the force-fields of water model involving four charge sites (TIP4P) and the optimized potentials for liquid simulations (OPLS-AA) and assigned with RESP charges.

Bingding energy. The density functional theory (DFT) calculations were performed using the Vienna Ab Initio Package (VASP) with the generalized gradient approximation Perdew-Burke-Ernzerhof (GGA-PBE) functional. Projected augmented wave (PAW) potentials[2-7]were chosen to describe the ionic cores, and valence electrons were described using plane wave basis set with a kinetic energy cutoff of 500 eV. The electronic energy was converged when the total energy change was smaller than 10^{-4} eV. The residual force threshold for the convergence of geometry optimization was set to be 10^{-2} eV Å. We model the surface using a symmetric periodic slab, (003) crystal face of NCM811, and a 15 $Å^{-2}$ vacuum layer was inserted between the slab and its periodic image. The atoms of matrix slab are fixed for reducing calculation only when evaluated adsorption energy.

The binding energy of individual molecules on the NCM811 surface were computed as:

$$E_b=E_{total}-E_{NCM811\_slab}-E_{Individual\ molecules} \quad (2)$$

Where E_{NCM811_slab} and $E_{Individual\ molecules}$ are the energy of the bare NCM811 surface and individual molecules, respectively, and E_{total} was the total energy of the configurations of individual molecule on the NCM811 surfaces.

Supplementary Figures

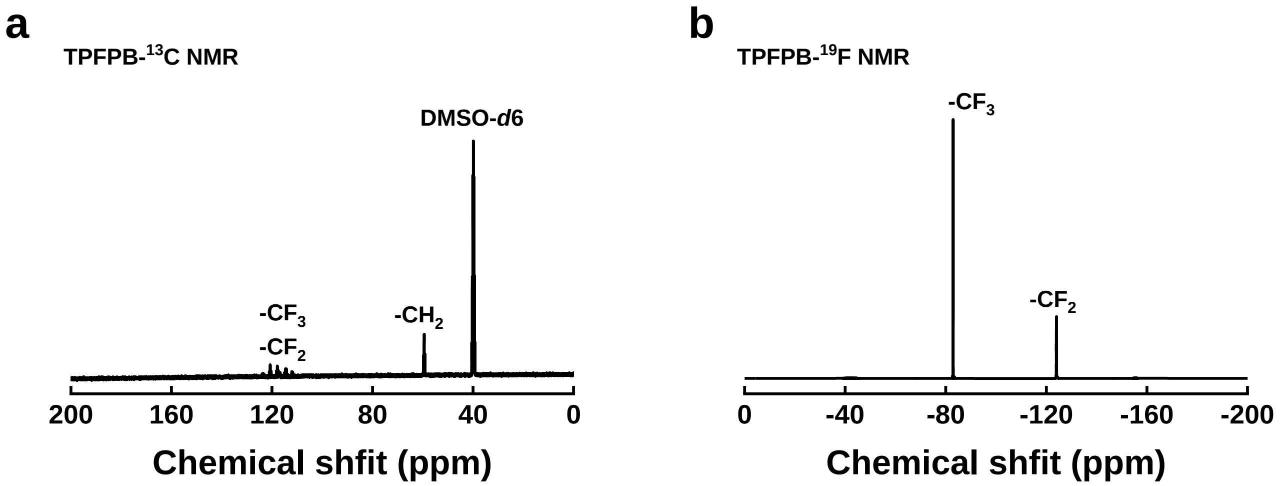

Figure S1. NMR characterization of TPFPB. (a) ^{13}C NMR and (b) ^{19}F NMR spectra of TPFPB in DMSO-d_6.

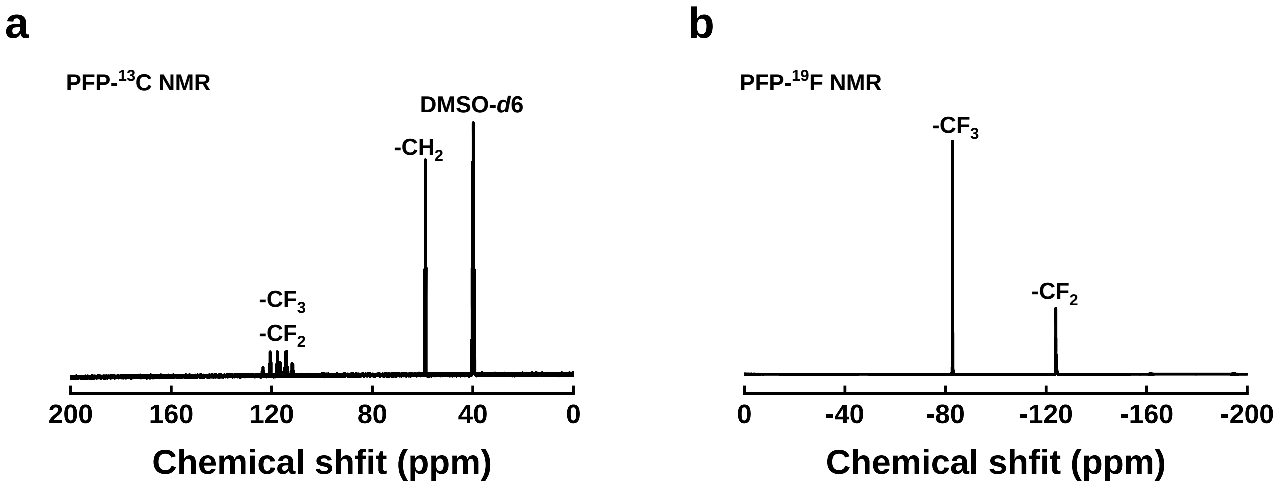

Figure S2. NMR characterization of PFP. (a) ^{13}C NMR and (b) ^{19}F NMR spectra of PFP in DMSO-d_{6}.

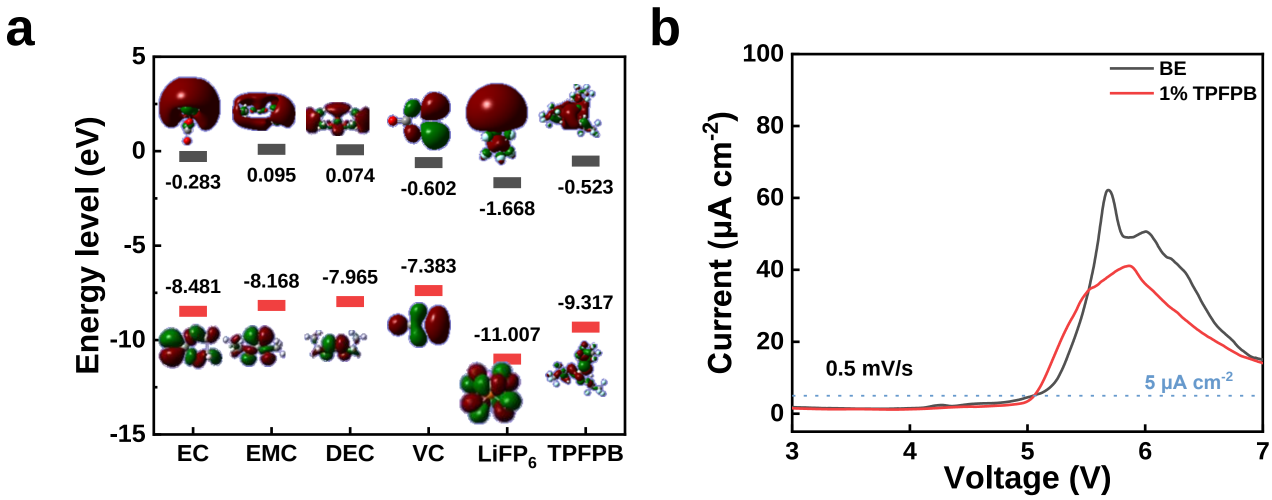

Fig. S3. (a) HOMO and LUMO energy levels of EC, EMC, DEC, VC, $LiPF_6$ and TPFPB. (b**)** Linear sweep voltammetry measurements of BE and 1% TPFPB at 25 ℃ under sweep rate of 0.5 mV s^{-1}.

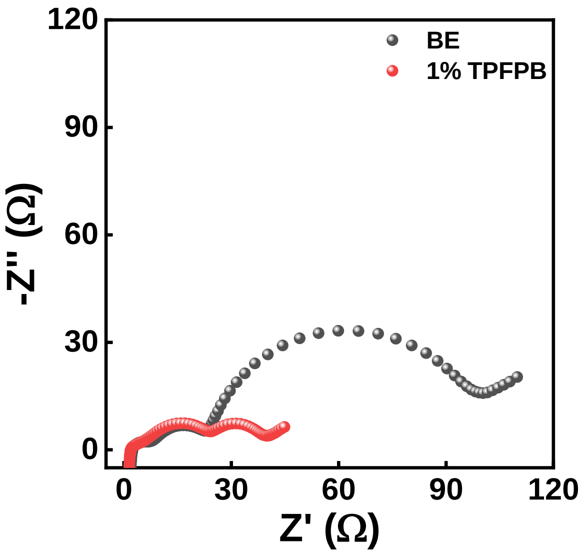

Figure S4. Electrochemical impedance spectra of Li||NCM811 cells after rate performance test at 25 ℃.

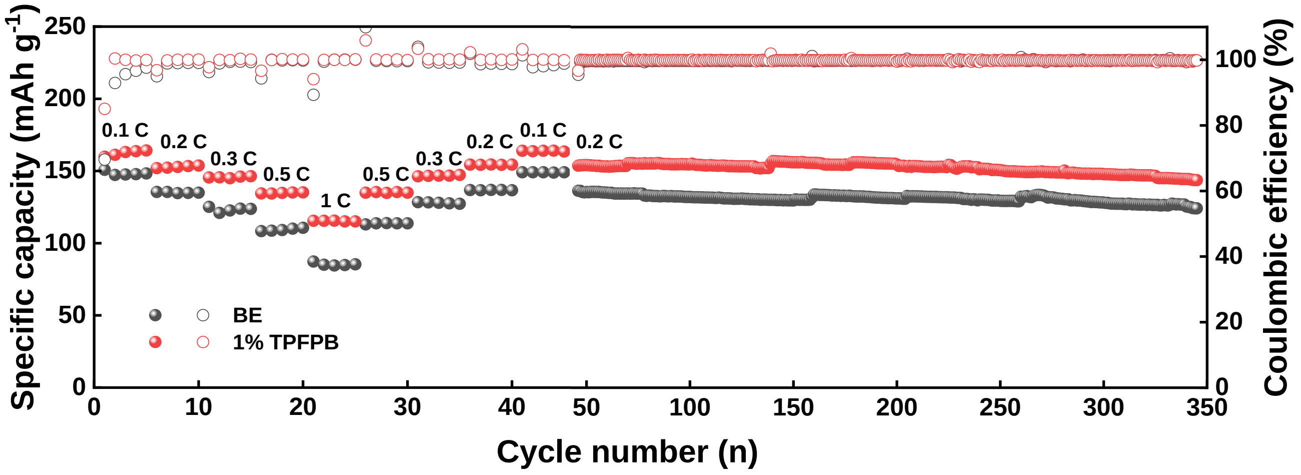

Figure S5. Cycling and rating performance of Li||NCM811 cells using BE and BE with 1%TPFPB at -20 ℃

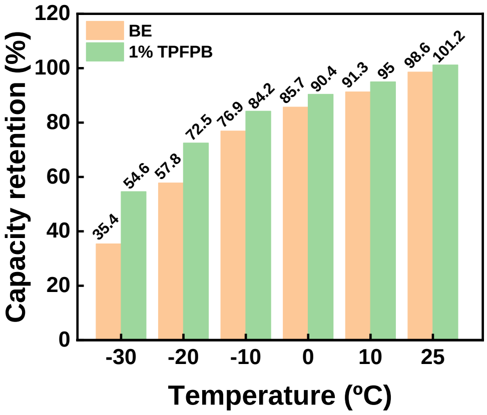

Figure S6. Capacity retention of Li||NCM811 cells at different temperature to RT capacity using BE and BE with 1%TPFPB

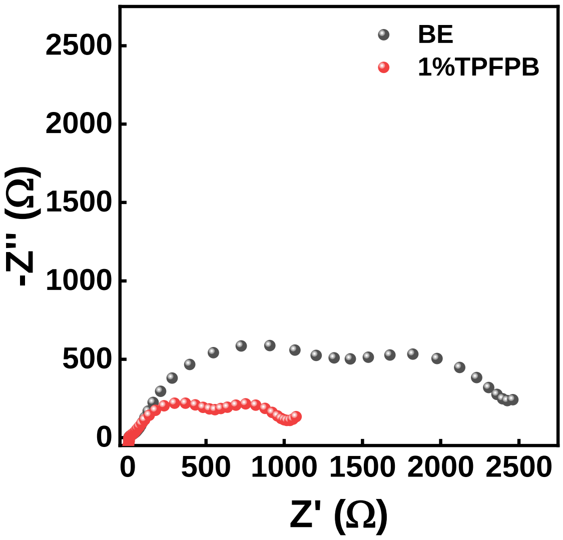

Figure S7. Electrochemical impedance spectra of Li||NCM811 cells using BE and BE with 1%TPFPB after 50 cycles at 0.2 C under -30 ℃.

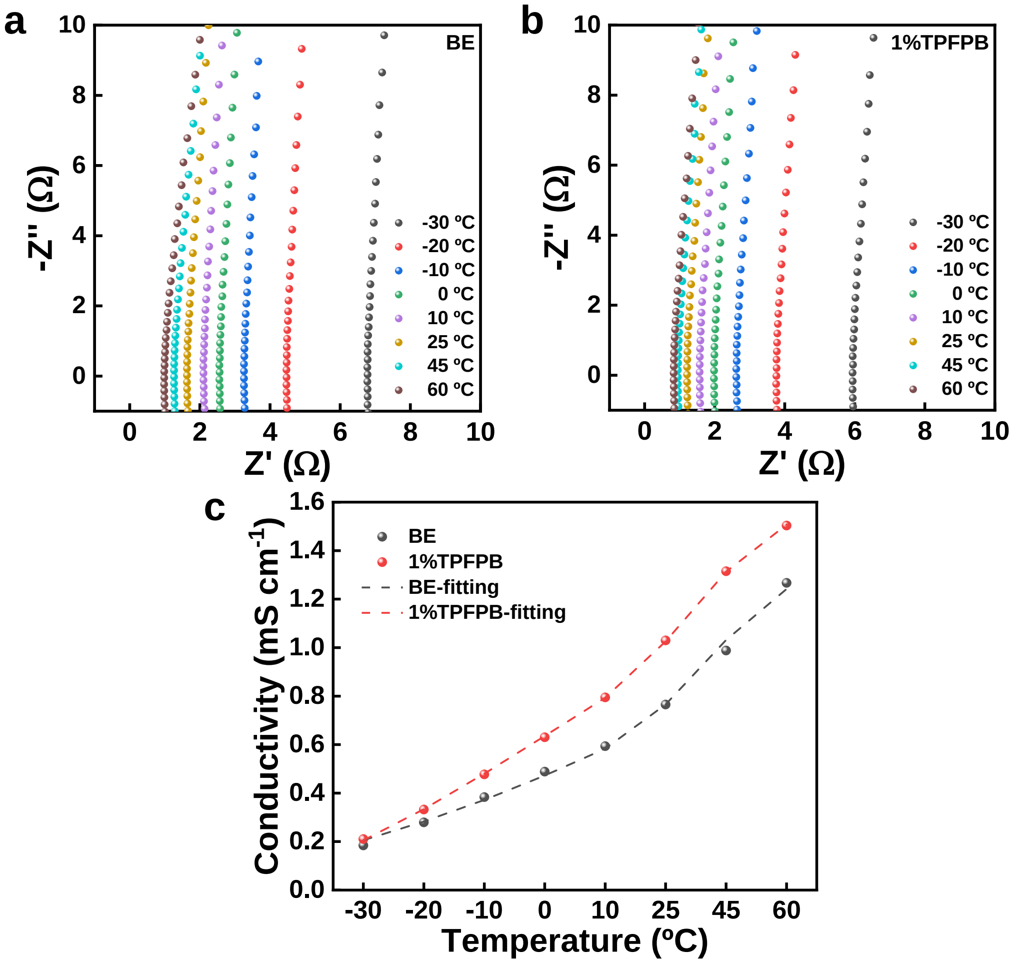

Figure S8. Electrochemical impedance spectra of stainless steel||stainless steel cells with (a) BE and (b) 1% TPFPB at different temperature. (c) VTF function fitting of relationship of ionic conductivity of BE and BE with 1% TPFPB at different temperatures.

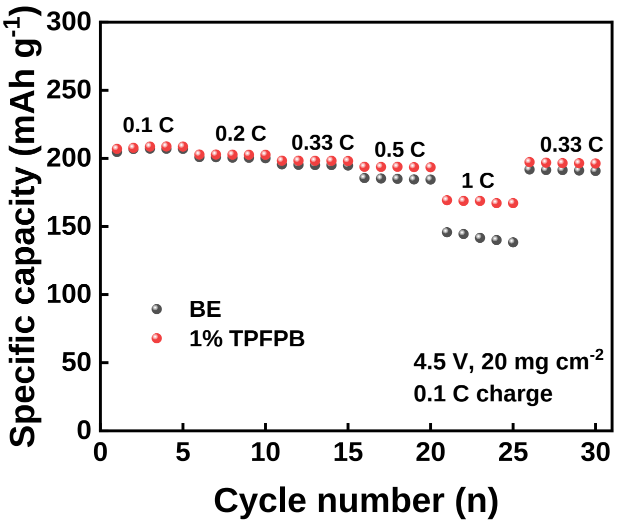

Figure S9. Rate performance of Li||NCM811 cells with high cathode loading of 20 mg cm^{-2} between 3 V and 4.5 V at 25 ℃.

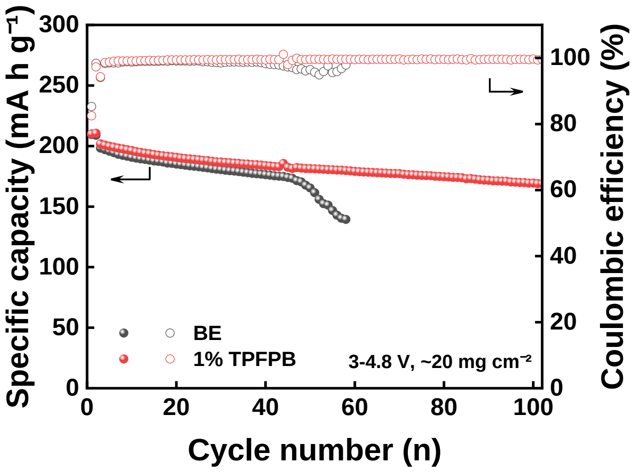

Figure S10. Cycling performance of thin Li||NCM811 coin cells at 25 ℃ between 3 and 4.8 V. The charge/discharge rate is 0.1/0.333 C, the electrolyte amount of cells is 40 μL and the N/P ratio of these cells is ~10.

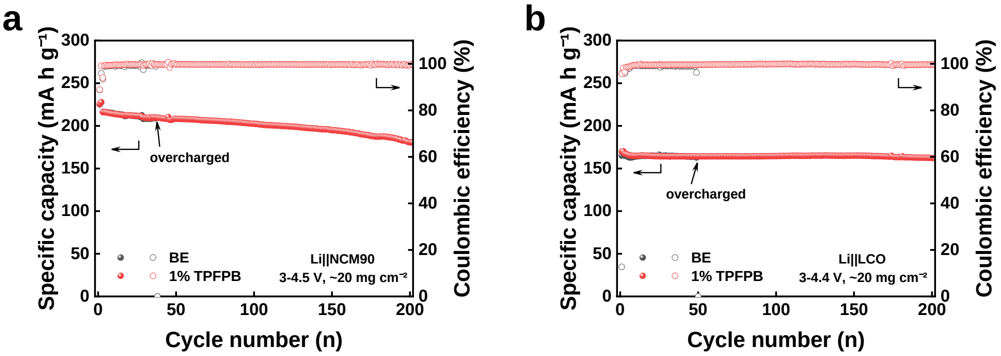

Figure S11. Cycling performance of (a) Li||NCM90 and (b) Li||LCO coin cells at 25 ℃. The charge/discharge rate is 0.1/0.333 C, the thickness of Li anode is around 200 μm and the N/P ratio of these cells is ~10.

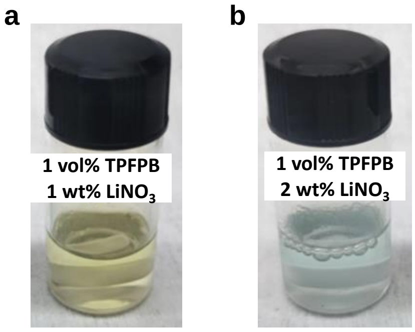

Figure S12. Photograph of carbonate electrolyte with 1 vol% TPFPB and (a) 1 wt % $LiNO_3$ and (b) 2 wt% $LiNO_3$.

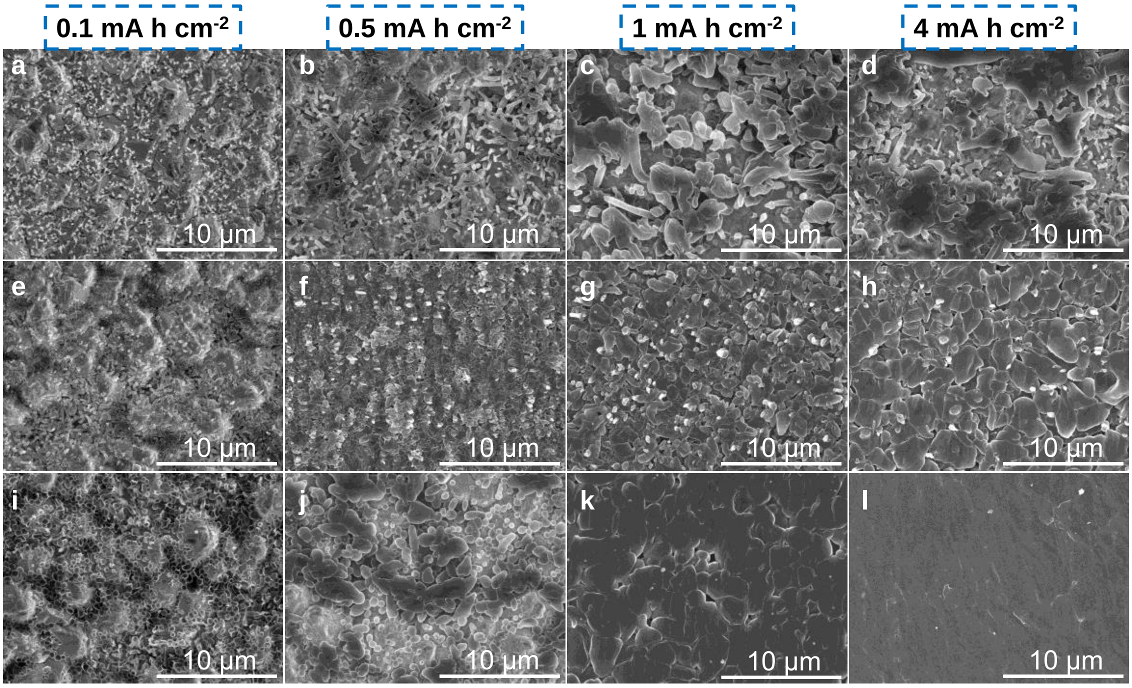

Figure S13. Surface morphology of lithium deposition on Cu electrode using BE and BE with 1% TPFPB at different areal capacity. (a, e, i) 0.1 mA h cm^{-2}; (b, f, j) 0.5 mA h cm^{-2}; (c, g, k) 1 mA h cm^{-2}; (d, h, l) 4 mA h cm^{-2}. (a-d) Li||Cu cell using BE; (e-h) Li||Cu cell using BE with 1% TPFPB; (i-j) Li||Cu cell using BE with 1%TPFPB-1%$LiNO_3$.

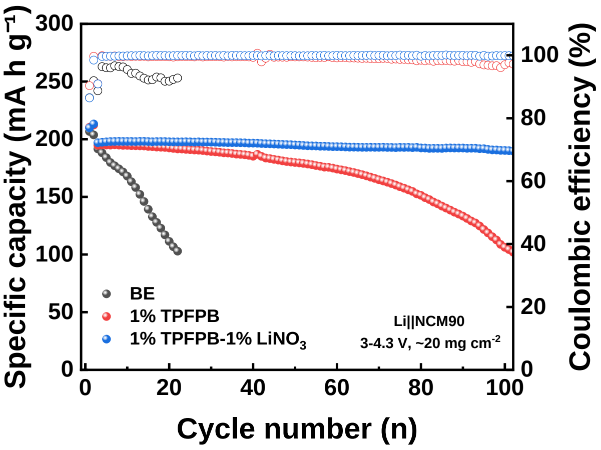

Figure S14. Cycling performance of Li||NCM90 coin cells between 3 and 4.3 V at 25 ℃. The charge/discharge rate is 0.5/1 C, the areal mass-loading of NCM90 is around 20 mg cm^{-2}, the thickness of Li anode is ~ 200 μm, and the N/P ratio of these cells is ~10.

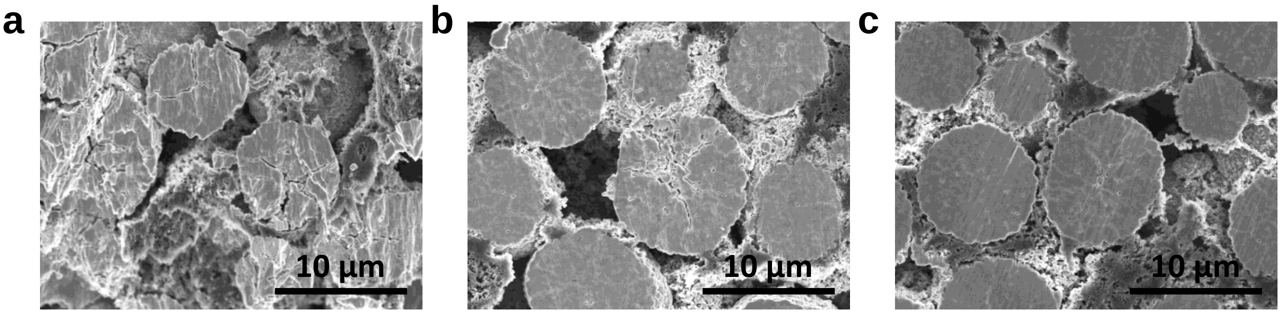

Figure S15. Cross section image of NCM90 cathode retrieved from (a) Li||NCM90 cell using BE, (b) Li||NCM90 cell using BE with 1% TPFPB and (c) Li||NCM cell using BE with 1% TPFPB and 1% $LiNO_3$ after 100 cycles between 3 and 4.3 V at 25 ℃. The charge/discharge rate is 0.5/1 C, the areal mass-loading of NCM90 is around 4 mA h cm^{-2}, the thickness of Li anode is ~ 200 μm and the N/P ratio of these cells is ~10.

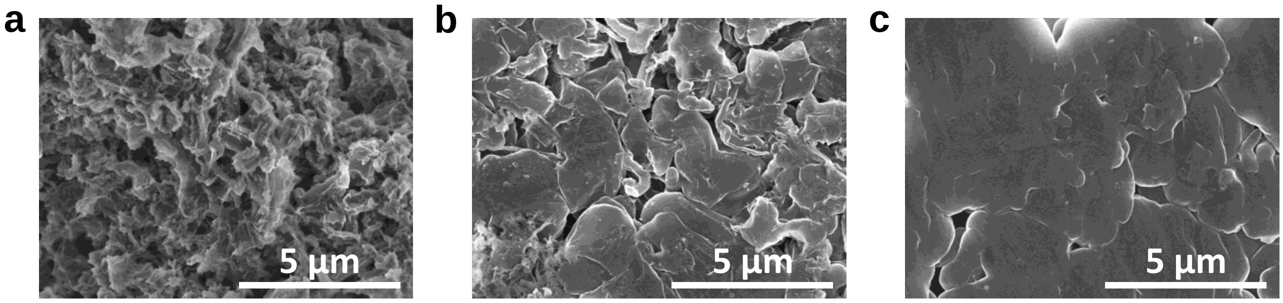

Figure S16. The surface morphology image of Li anode retrieved from (a) Li||NCM90 cell using BE, (b) Li||NCM90 cell using BE with 1% TPFPB and (c) Li||NCM cell using BE with 1% TPFPB and 1% $LiNO_3$ after 100 cycles between 3 and 4.3 V at 25 ℃. The charge/discharge rate is 0.5/1 C, the areal mass-loading of NCM90 is around 4 mA h cm^{-2}, the thickness of Li anode is ~ 200 μm and the N/P ratio of these cells is ~10.

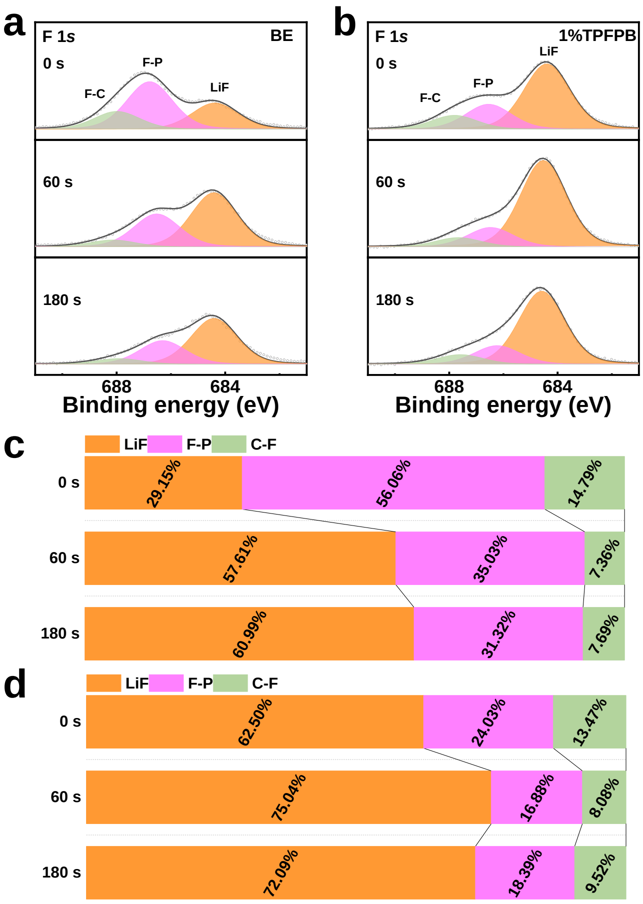

Figure S17. Ex situ XPS measurements and analysis of NCM811 cathode retrieved from (a, c) Li||NCM811 cells using BE and (b, d) Li||NCM811 cells using BE with 1% TPFPB after 5 cycles at 0.1 C. The XPS spectra of (a, b) F 1s and (c, d) its corresponding composition and distribution of different constituents. The cells were disassembled at fully discharged state.

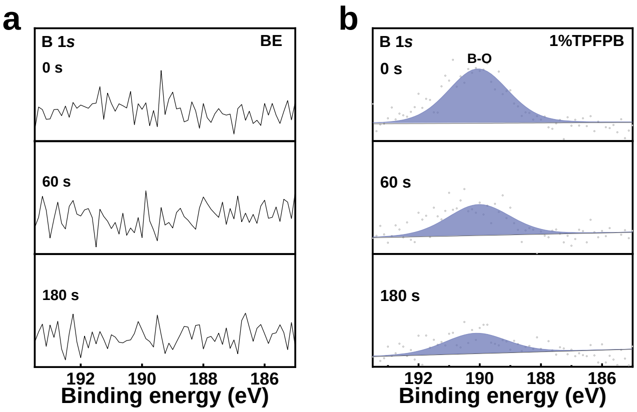

Figure S18. Ex situ XPS spectra of B 1s of NCM811 cathode retrieved from (a) Li||NCM811 cells using BE and (b) Li||NCM811 cells using BE with 1% TPFPB after 5 cycles at 0.1 C. The cells were disassembled at fully discharged state. The lithium salt of electrolyte is $LiClO_4$.

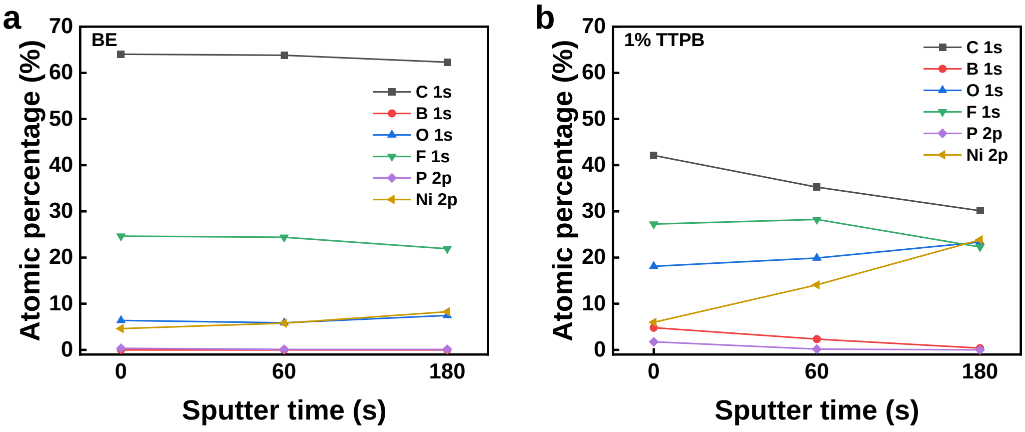

Figure S19. Ex situ XPS depth profiles of NCM811 cathode retrieved from (a) Li||NCM811 cells using BE and (b) Li||NCM811 cells using BE with 1% TPFPB after 5 cycles at 0.1 C.

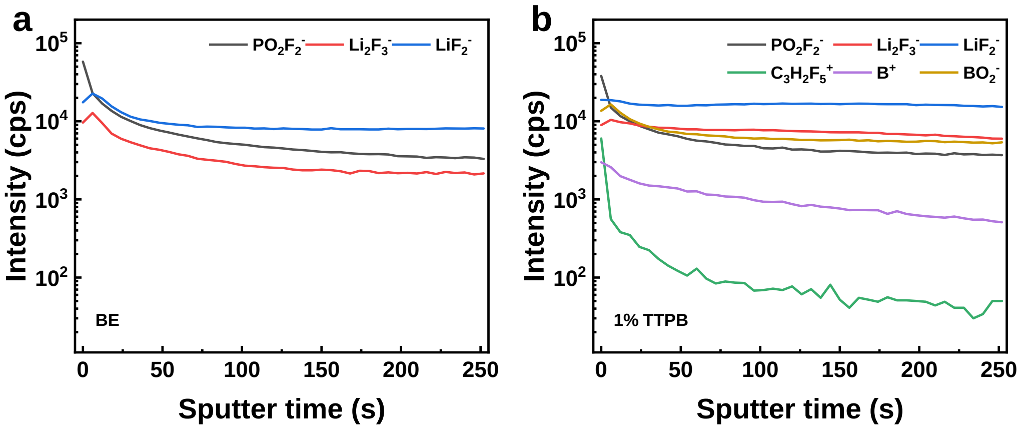

Figure S20. Depth profiling of secondary ion fragments on the NCM811 surface using (a) BE and (b) BE with 1% TPFPB after 5 cycles at 0.1 C under 25 °C.

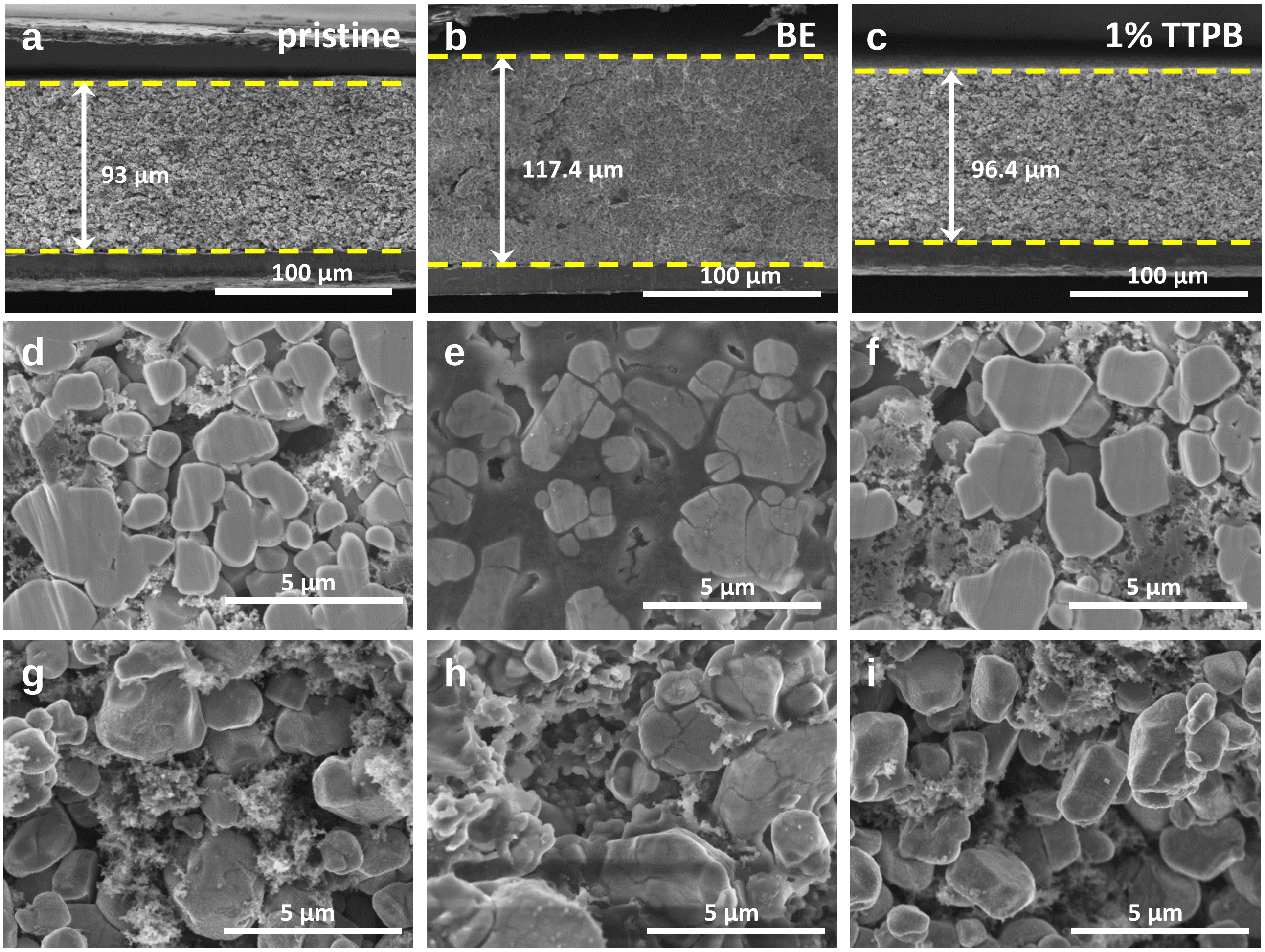

Figure S21. Cross section image of (a, d) pristine NCM811, (b, e) NCM811 using BE and (c, f) NCM811 using TPFPB. Surface morphology of (g) pristine NCM811, (h) NCM811 using BE and (i) NCM811 using TPFPB after 100 cycles at 25 °C. Charging/discharging rare is 0.1/0.333 C and the mass-loading of cathode is 4 mA h cm^{-2}.

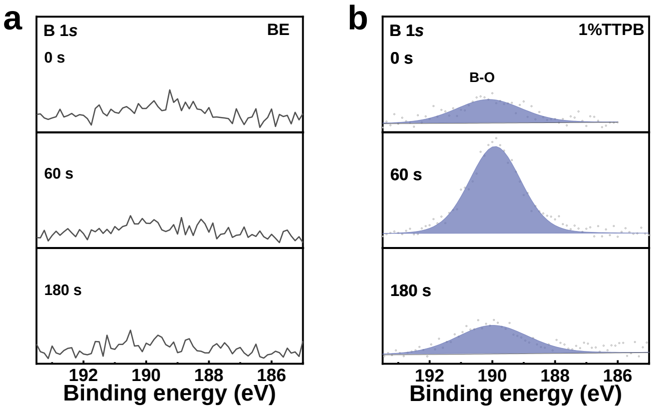

Figure S22. Ex situ XPS spectra of B 1s of LMA retrieved from (a) Li||NCM811 cells using BE and (b) Li||NCM811 cells using BE with 1% TPFPB after 5 cycles at 0.1 C. The cell was disassembled at fully discharged state. The lithium salt of electrolyte is $LiClO_4$.

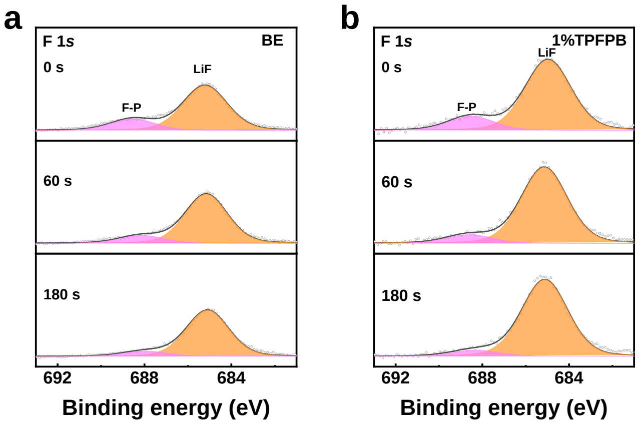

Figure S23. Ex situ XPS spectra of F 1s of LMA retrieved from (a) Li||NCM811 cells with BE and (b) Li||NCM811 cells with 1% TPFPB after 5 cycles at 0.1 C. The cell was disassembled at fully discharged state.

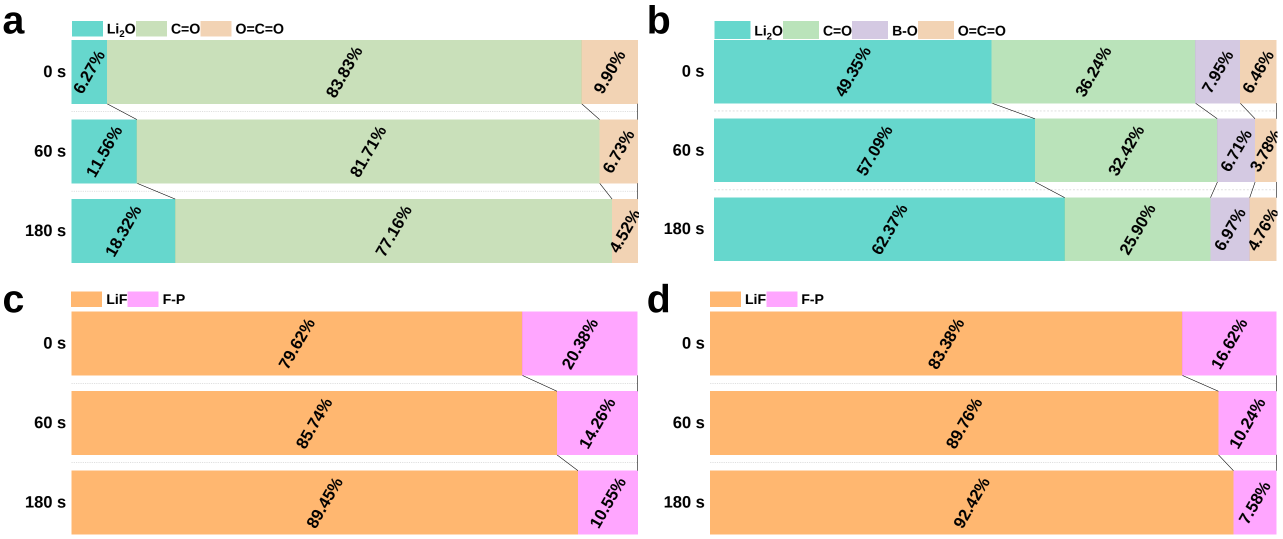

Figure S24. Composition and distribution of different constituents of SEI on LMA retrieved from Li||NCM811 cells using (a, c) BE and (b, d) BE with 1% TPFPB obtained from XPS of (a, b) O 1*s* and (c, d) F 1*s*.

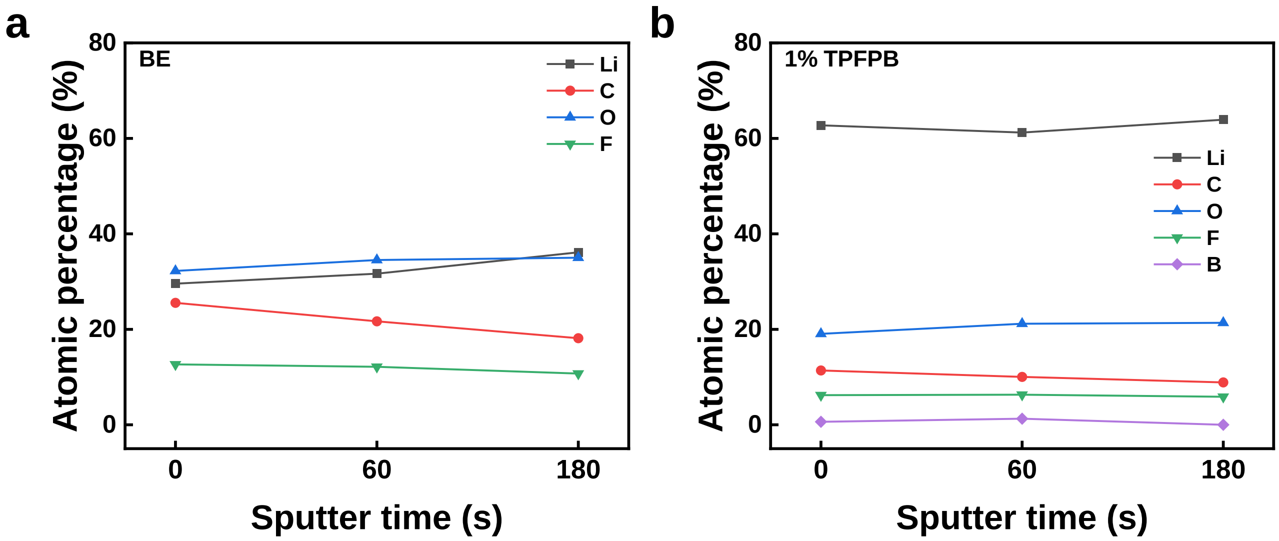

Figure S25. Ex situ XPS depth profiles of LMA retrieved from (a) Li||NCM811 cells using BE and (b) Li||NCM811 cells using BE with 1% TPFPB after 5 cycles at 0.1 C.

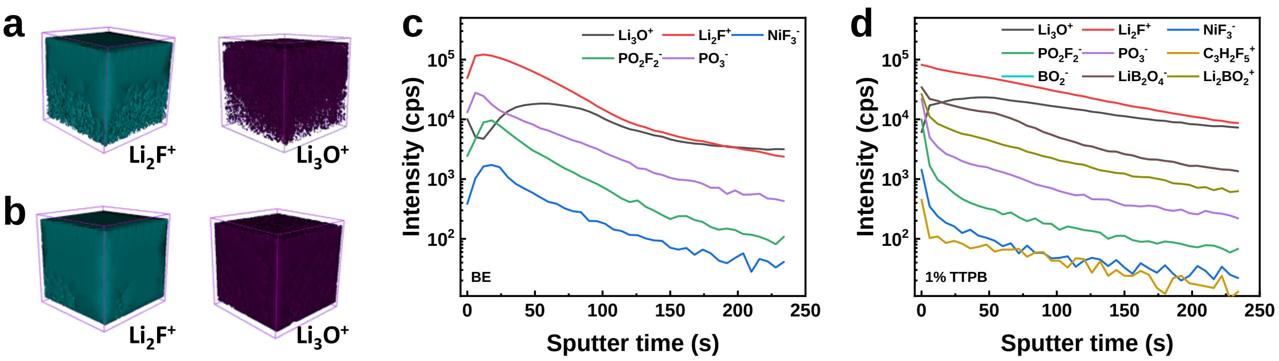

Figure S26. Ex situ TOF-SIMS 3D reconstruction of the sputtered volume on the cycled NCM811 surface using BE (a) and BE with 1% TPFPB (b). Depth profiling of several secondary ion fragments on the NCM811 surface using BE (c) and BE with 1% TPFPB (d) after 5 cycles at 0.1 C under 25 °C.

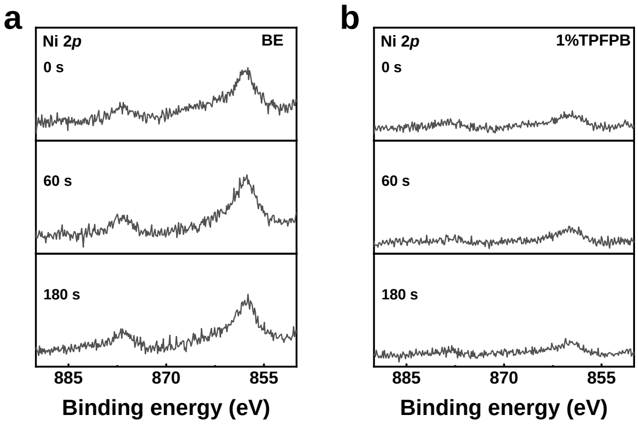

Figure S27. Ex situ XPS spectra of Ni 2p of LMA retrieved from (a) Li||NCM811 cells using BE and (b) Li||NCM811 cells using BE with 1% TPFPB after 5 cycles at 0.1 C. The cells were disassembled at fully discharged state.

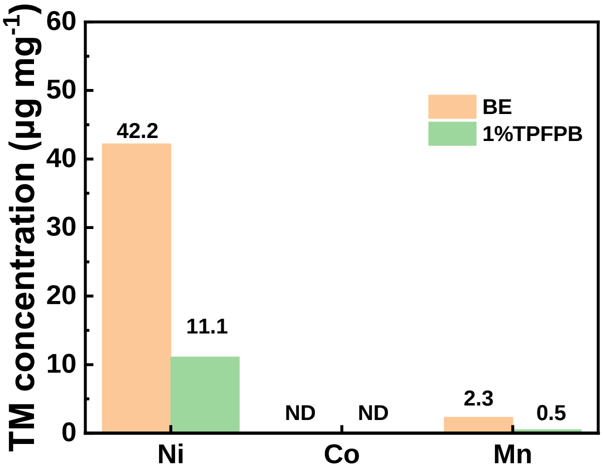

Figure S28. ICP-OES analysis of cycled LMA after 5 cycles at 0.1 C under 25 °C. The cells were disassembled at fully discharged state.

Supplementary Table

Table S1. Electrochemical performance comparation of Li||NCM cells with recently reported state-of-the-art electrolytes.

Battery type	Electrolyte	Anode	Cathode	N/P ratio	Electrolyte amount	E/C ratio	Cut-off voltage	Cathode mass-loading	Capacity	Energy density	Cycling condition & capacity retention	Ref
Coin cell	1 M $LiPF_6$ EC/DEC/EMC+ VC+1% TPFPB	Li	NCM811	10<br>6.7<br>10	40 µL	NM	4.5 V<br>4.5 V<br>4.8 V	4 mA h cm^{-2}<br>6 mA h cm^{-2}<br>4 mA h cm^{-2}	NM	NM	85.8% (300 cycles at 0.1/1/3 C)<br>90.6% (100 cycles at 0.1/1/3 C)<br>83.6% (100 cycles at 0.1/1/3 C)	This work
Coin cell	LiFSI-1.2DME-3TTE	Li	NCM811	2.2	14 µL	3 g $(Ah)^{-1}$	4.4 V	4.2 mA h cm^{-2}	NM	325 W h kg^{-1}, excluding con-cell parts	80% (155 cycles at 1/3/1/3 C)	[8]
Coin cell	2.5 M LiFSI + 0.2 M LiPF6-FSA	Li	NCM622	7.6	40 µL	NM	4.3 V	1.6 mA h cm^{-2}	NM	NM	89% (200 cycles at 1/3/1/3 C)	[9]
Coin cell	1 M	Li	NCM811	7.06	80 µL	NM	4.7 V	~1.7 mA h	NM	NM	88.1% (100 cycles at 0.5/0.5 C)	[10]

	LiFSI-DMTMSA							cm^{-2}				
Coin cell	1 M LiFSI-DME/TFEO	Li	NCM811	7.07	75 μL	NM	4.4 V	1.5 mA h cm^{-2}	NM	NM	80% (300 cycles at 1/3/1/3 C)	[11]
Coin cell	LiPF6-EC/DEC-LiNO3-$Sn(OTf)_2$	Li	NMC811	2.64	NM	10 μL mAh^{-1}	4.3 V	16.7 mg cm^{-2}	NM	NM	89.6% (130 cycles at 0.3/0.3 C)	[12]
Coin cell	2 M LiFSI-TFDMP	Li	NMC811	1	NM	NM	4.4 V	20 mg cm^{-2}	NM	NM	81% (200 cycles at 0.1/ 0.1 A g^{-1})	[13]
Coin cell	1 M LiPF6 FEC-EMC +2wt% LiBF4+2wt% LiNO3	Li	NCM811	3.86	40 μL	NM	4.4 V	2.4 mA h cm^{-2}	NM	NM	80.3% (250 cycles at 0.3/0.5 C)	[14]
Coin cell	1 M LiPF6/FEC-EMC, LiNO3-TPFPB	Li	NMC811	2.3	NM	~3.4 g $(Ah)^{-1}$	4.5 V	4 mAh cm^{-2}	NM	NM	75.4% (100 cycles at 0.2/0.3 C)	[15]
Coin cell	1M LiTFSI-FEC/FDMA	Li	NCM811	1.5	NM	30 g $(Ah)^{-1}$	4.3 V	3.5 mA h cm^{-2}	NM	NM	92% (100 cycles at 0.25/0.25 C)	[16]

Pouch cell	1 M $LiPF_6$ EC/DEC/EMC+ VC+1% TPFPB+1% $LiNO_3$	Li	NCM90	3.1	NM	1.4 g $(Ah)^{-1}$	4.3 V	3.2 mA h cm^{-2}	3.84 A h	420.96 W h kg^{-1} at 0.1 C 383.1 W h kg^{-1} at 1 C	94.88% (100 cycles at 0.1/0.5 C)	This work
Pouch cell	1 M LiFSI-DMTMSA	Li	NCM811	2.9	NM	2.3 g $(Ah)^{-1}$	4.7 V	18.4 mg cm^{-2}	NM	353 W h kg^{-1}	~75 % (100 cycles at 0.2/0.5 C)#	[10]
Pouch cell	5 M LiFSI+0.16 M NaTFSI -EMImFSI	Li@Cu	NCM811	1.8	0.3 mL	NM	4.4 V	10 mg cm^{-2}	NM	NM	95% (120 cycles at 0.5/0.5 C)	[17]
Pouch cell	2 M LiFSI-BFE		NCM811	2	NM	2.4g $(Ah)^{-1}$	4.4 V	4 mA h cm^{-2}	NM	NM	> 80%(200 cycle at 0.8/8 $mAcm^{-2}$)	[18]
Pouch cell	1.2 M LiFSI/TEP-BTFE	Li (50 μm)	NCM622	2.6	NM	3 g $(Ah)^{-1}$	4.4 V	3.8 mA h cm^{-2}	1.17 A h	313 W h kg^{-1}	86% (200 cycles at 0.1/0.333 C)	[19]
Pouch cell	1 M LiPF6+0.05 M LiDFOB/EC-DEC-DMC-FEC +10.5wt% porous LiF nanobox	Li (50 μm)	NCM811	2.5	NM	2.9 g $(Ah)^{-1}$	4.3 V	NM	3.45 A h	380 W h kg^{-1}	93% (86 cycles at 0.2/0.5 C)	[20]

Pouch cell	0.6M LiTFSI+0.4 M LiBOB+0.4 M LiF+0.1M LiNO3+0.05M LiPF6+0.03 M LiBF4/EC-DMC +1 wt% FEC+3wt% TFEC	Li (100 μm)	NCM811	~5	300 μL	NM	4.3 V	4.1 mA h cm^{-2}	~0.2 A h	260 W h kg^{-1} without counting tap and packing foil	~85% (120 cycles at 0.1/1 C)	[21]
Pouch cell	1 M LiPF6/EC-DMC+2wt% LiDFP+TTE	Li (45 μm)	NCM811	2.66	NM	2.82	4.3 V	19.5 mg cm^{-2}	3.2 A h	368 W h kg^{-1}	62.5% (50 cycles at 0.1/0.1 C)	[22]
Pouch cell	1.2M LiFSI/F5DEE	Li (25 μm)	NCM811	1.32	NM	~2.5	4.4 V	3.8 mA h cm^{-2}	0.12 A h	NM	85% (150 cycles at 0.2/0.5 C)	[23]
Coin cell	1.2M LiFSI/F5DEE	Li (50 μm)	NCM811	2.04	NM	8	4.4 V	4.9 mA h cm^{-2}	NM	NM	80% (270 cycles at 0.1/0.3 C)	[23]

Pouch cell	1.5 M LiPF6/FEC-EMC-DMC)+1wt%TMSB	Li	NCM811	2.54	NM	3g (Ah)$^{-1}$	4.3 V	5.12 mA h cm^{-2}	2.5 A h	357.2 W h kg^{-1}	90.9% (50 cycles at 0.2/0.2 C)	[24]
Pouch cell	1.5 M DME-TTE	Li (20 μm)	NCM622	1	NM	2.4 g (Ah)$^{-1}$	4.4 V	4.0 mA h cm^{-2}	2 A h	350 W h kg^{-1}	76% (600 cycles at 0.1/0.3 C)	[25]
Pouch cell	1 M LiPF6 +0.1 M LiFEA+0.1 M $LiNO_3$/EC-DEC	Li (50 μm)	NCM811	2.7	NM	2.8 g (Ah)$^{-1}$	4.3 V	3.66 mA h cm^{-1}	0.42 A h	310 W h kg^{-1}	90% (120 cycles at 0.2/0.6 C)	[26]
Pouch cell	1 M LiPF6 +1 M LiTFSI+0.1 M LiDFBOP/ FEC-EMC	Li (42.5 μm)<br>Li (50 μm)	NCM83	1.9<br>2	NM	2.0 g (Ah)$^{-1}$<br>1.2 g (Ah)$^{-1}$	4.3 V<br>4.4 V	4.5 mA h cm^{-1}<br>5.2 mA h cm^{-1}	2 Ah<br>6 Ah	401.8 W h kg^{-1}<br>472.4 W h kg^{-1}	95.6% (80 cycles at 0.2/0.2 C)<br>81.5% (150 cycles at 0.1/0.5 C)	[27]

the article doesn’t mentioned the capacity retention of this pouch cell. NM present Not mentioned.

Table S2. Detailed parameters of 3.1 Ah Li||NCM90 pouch cells.

	Parameter	Value
NCM90 cathode	Area weight	30.25 mg cm^{-2}
	Number of layers	17
Al foil	Thickness	15 μm
Li anode	Li thickness	100 μm
Separator	Type	LATP-coated polyethylene
	Thickness	10 μm
Electrolyte	E/C ratio	1.4 g $(A\ h)^{-1}$
Package foil	Thickness	113 μm
Pouch cell	Weight	34.96 g
	Discharge capacity	3.838 A h (0.1 C)<br>14.716 W h (0.1 C)
		3.6252 A h (0.5 C)<br>13.834 W h (0.5 C)
	Energy density	420.96 W h kg^{-1} (0.1 C)
		395.71 W h kg^{-1} (0.5 C)

Note: the area weight of NCM90 cathode is the total weight of cathode, which is included in Al foil, cathode particle, super P and PVDF.

Supplementary References

1. Quartarone E and Mustarelli P. Electrolytes for solid-state lithium rechargeable batteries: recent advances and perspectives. *Chemical Society Reviews*. 2011; **40**: 2525-40.
2. Blöchl PE. Projector augmented-wave method. *Phys Rev B*. 1994; **50**: 17953-79.
3. Kresse G and Hafner J. Ab initio molecular dynamics for liquid metals. *Phys Rev B*. 1993; **47**: 558-61.
4. Kresse G and Hafner J. Ab initio molecular-dynamics simulation of the liquid-metal-amorphous-semiconductor transition in germanium. *Phys Rev B*. 1994; **49**: 14251-69.
5. Kresse G and Furthmüller J. Efficiency of ab-initio total energy calculations for metals and semiconductors using a plane-wave basis set. *Comput Mater Sci*. 1996; **6**: 15-50.
6. Kresse G and Furthmüller J. Efficient iterative schemes for ab initio total-energy calculations using a plane-wave basis set. *Phys Rev B*. 1996; **54**: 11169-86.
7. Perdew JP, Burke K and Ernzerhof M. Generalized gradient approximation made simple. *Phys Rev Lett*. 1996; **77**: 3865-8.
8. Ren X, Zou L, Cao X, *et al.* Enabling high-voltage lithium-metal batteries under practical conditions. *Joule*. 2019; **3**: 1662-76.
9. Xue W, Shi Z, Huang M, *et al.* FSI-inspired solvent and “full fluorosulfonyl” electrolyte for 4 V class lithium-metal batteries. *Energy Environ Sci*. 2020; **13**: 212-20.
10. Xue W, Huang M, Li Y, *et al.* Ultra-high-voltage Ni-rich layered cathodes in practical Li metal batteries enabled by a sulfonamide-based electrolyte. *Nat Energy*. 2021; **6**: 495-505.
11. Cao X, Ren X, Zou L, *et al.* Monolithic solid–electrolyte interphases formed in fluorinated orthoformate-based electrolytes minimize Li depletion and pulverization. *Nat Energy*. 2019; **4**: 796-805.
12. Zhang W, Wu Q, Huang J, *et al.* Colossal granular lithium deposits enabled by the grain-coarsening effect for high-efficiency lithium metal full batteries. *Adv Mater*. 2020; **32**: e2001740.
13. Zhao Y, Zhou T, Mensi M, *et al.* Electrolyte engineering via ether solvent fluorination for developing stable non-aqueous lithium metal batteries. *Nat Commun*. 2023; **14**: 299.
14. Wang X, Li S, Zhang W, *et al.* Dual-salt-additive electrolyte enables high-voltage lithium metal full batteries capable of fast-charging ability. *Nano Energy*. 2021; **89**: 106353.
15. Li S, Zhang W, Wu Q, *et al.* Synergistic dual-additive electrolyte enables practical lithium-metal batteries. *Angew Chem Int Ed*. 2020; **59**: 14935-41.
16. Wang Q, Yao Z, Zhao C, *et al.* Interface chemistry of an amide electrolyte for highly reversible lithium metal batteries. *Nat Commun*. 2020; **11**: 4188.
17. Sun H, Zhu G, Zhu Y, *et al.* High-safety and high-energy-density lithium metal batteries in a novel ionic-liquid electrolyte. *Adv Mater*. 2020; **32**: 2001741.
18. Zhang G, Chang J, Wang L, *et al.* A monofluoride ether-based electrolyte solution for fast-charging and low-temperature non-aqueous lithium metal batteries. *Nat Commun*. 2023; **14**: 1081.
19. Niu C, Lee H, Chen S, *et al.* High-energy lithium metal pouch cells with limited anode swelling and long stable cycles. *Nat Energy*. 2019; **4**: 551-9.
20. Tan Y-H, Lu G-X, Zheng J-H, *et al.* Lithium fluoride in electrolyte for stable and safe lithium-metal batteries. *Adv Mater*. 2021; **33**: 2102134.
21. Kim MS, Ryu J-H, Deepika, *et al.* Langmuir–Blodgett artificial solid-electrolyte interphases for practical lithium metal batteries. *Nat Energy*. 2018; **3**: 889-98.
22. Deng W, Dai W, Zhou X, *et al.* Competitive solvation-induced concurrent protection on the anode and cathode toward a 400 Wh kg^{-1} lithium metal battery. *ACS Energy Lett*. 2021; **6**: 115-23.

23. Yu Z, Rudnicki PE, Zhang Z, *et al.* Rational solvent molecule tuning for high-performance lithium metal battery electrolytes. *Nat Energy*. 2022; **7**: 94-106.
24. Huang K, Bi S, Kurt B, *et al.* Regulation of SEI formation by anion receptors to achieve ultra-stable lithium-metal batteries. *Angew Chem Int Ed*. 2021; **60**: 19232-40.
25. Niu C, Liu D, Lochala JA, *et al.* Balancing interfacial reactions to achieve long cycle life in high-energy lithium metal batteries. *Nat Energy*. 2021; **6**: 723-32.
26. Xia Y, Zhou P, Kong X, *et al.* Designing an asymmetric ether-like lithium salt to enable fast-cycling high-energy lithium metal batteries. *Nat Energy*. 2023.
27. Su H, Chen Z, Li M, *et al.* Achieving practical high-energy-density lithium-metal batteries by a dual-anion regulated electrolyte. *Adv Mater*. 2023; **35**: 2301171.
